# Facile Mechanophore Integration in Heterogeneous Biologically Derived Materials via “Dip-Conjugation”

**DOI:** 10.1021/jacs.4c03534

**Published:** 2024-06-20

**Authors:** Yifan Liao, Baptiste Le Roi, Hang Zhang, Charles E. Diesendruck, Joshua M. Grolman

**Affiliations:** † Materials Science and Engineering Department, Technion-Israel Institute of Technology, Haifa 3200003, Israel; ‡ Shulich Faculty of Chemistry, Technion-Israel Institute of Technology, Haifa 3200003, Israel

## Abstract

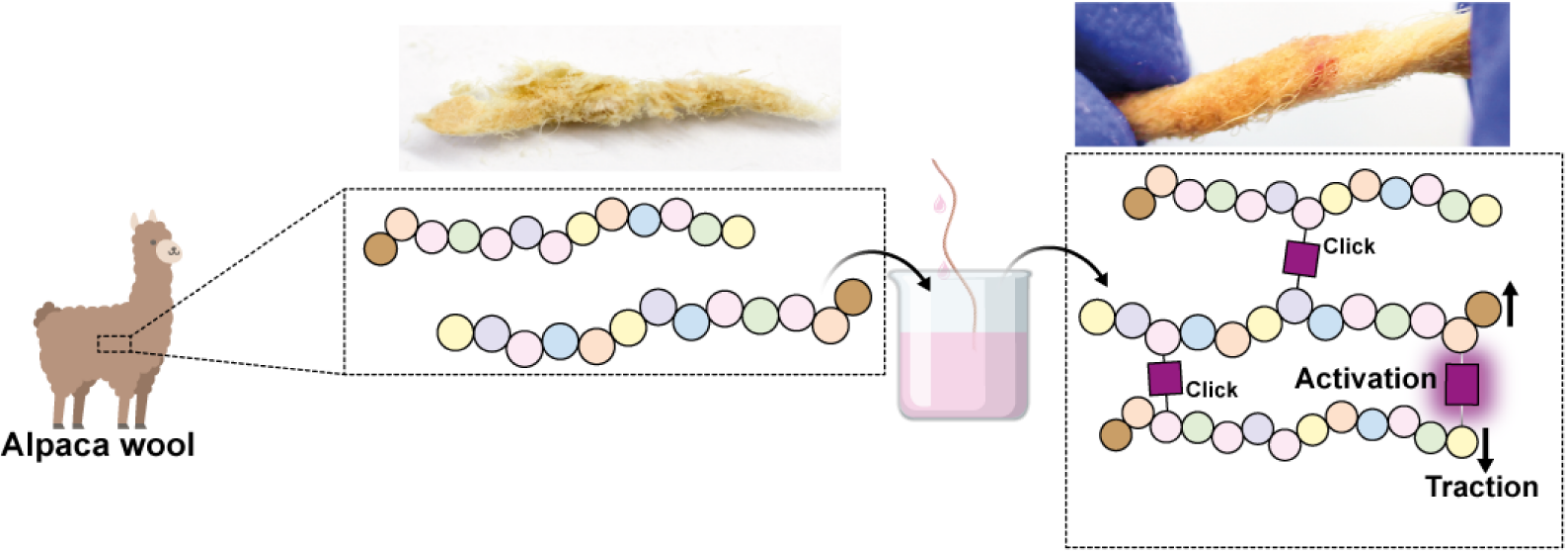

Mechanical forces play critical roles in a wide variety of biological processes and diseases, yet measuring them directly at the molecular level remains one of the main challenges of mechanobiology. Here, we show a strategy to “Dip-conjugate” biologically derived materials at the chemical level to mechanophores, force-responsive molecular entities, using Click-chemistry. Contrary to classical prepolymerization mechanophore incorporation, this new protocol leads to detectable mechanochromic response with as low as 5% strain, finally making mechanophores relevant for many biological processes that have previously been inaccessible. Our results demonstrate the ubiquity of the technique with activation in synthetic polymers, carbohydrates, and proteins under mechanical force, with alpaca wool fibers as a key example. These results push the limits for mechanophore use in far more types of polymeric materials in applications ranging from molecular-level force damage detection to direct and quantitative 3D force measurements in mechanobiology.

## Introduction

Mechanochemical transduction has been a dream of biomimicry for many years in the making. Where natural systems have evolved adept mechanisms to convert mechanical force in a diverse range of materials into biological readouts, our synthetic efforts have fallen short. On this front, fundamental studies on molecular-level force effects,^1^ and applications such as detecting material damage,^2,3^ drug delivery,^4^ and even self-healing plastics,^5^ all have benefited from using such molecules exhibiting chemical and detectable changes as a function of applied force. And yet, there is such a large appeal for molecular force sensor use in biological-derived materials, particularly in the field of mechanobiology where the links between development, disease, and mechanics are investigated. The importance of cells sensing their environment^6,7^ and also directing disease progression such as in myelofibrosis^8^ has been shown, though the challenges of molecular force sensor incorporation, which would allow us to connect between mechanical and biochemical processes, remain unsolved. Widespread use of force-sensitive molecules like mechanophores is impeded by both the difficulty in their integration into different bulk materials, particularly when chemistry and processing are separated,^9,10^ and low sensitivity, requiring very large strains for activation.

**Figure 1:**
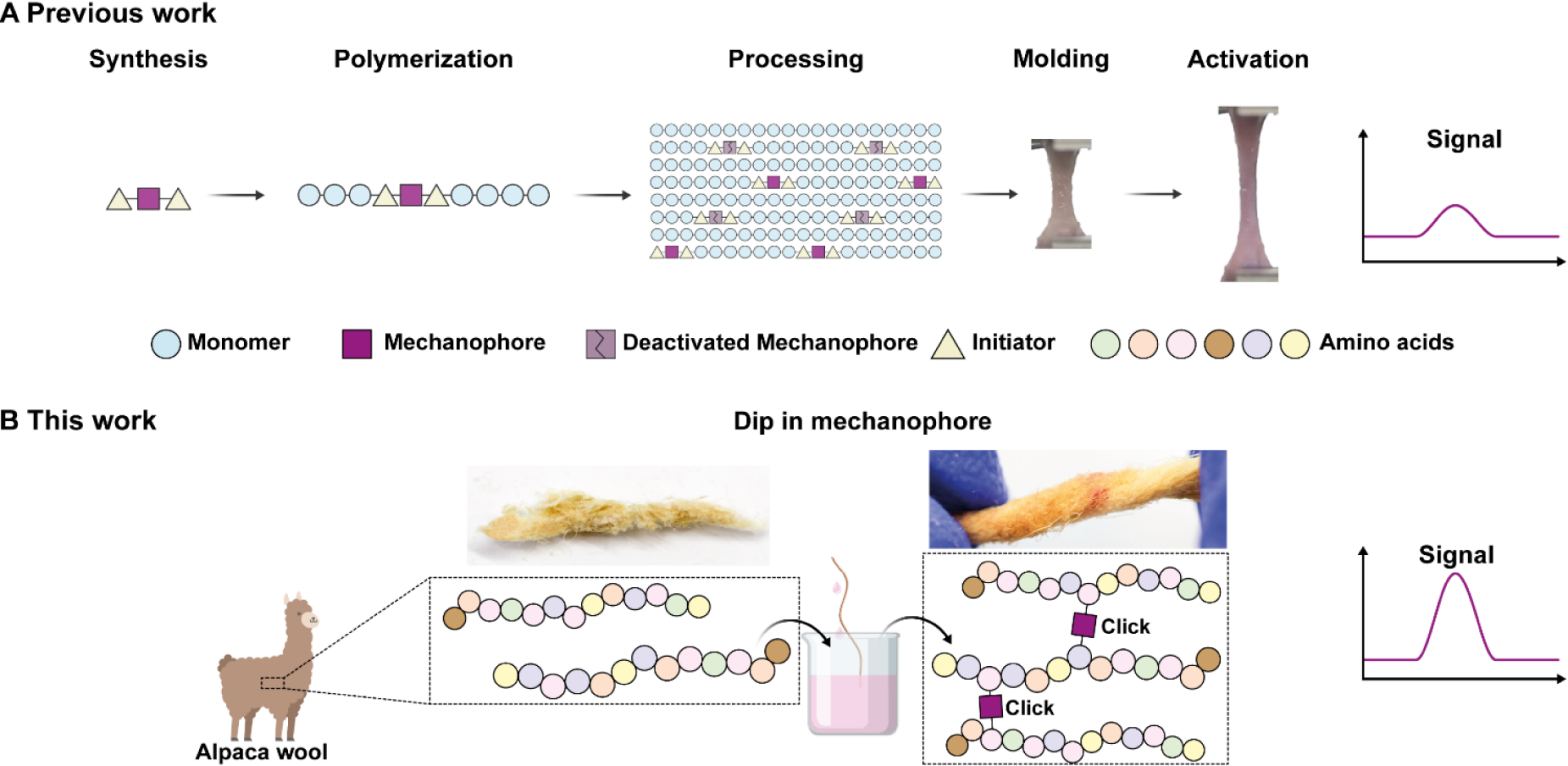
Schematic of the Click-chemistry-enabled Dip-conjugation functionalization strategy, enabling the incorporation of norbornene-decorated spiropyran derivatives onto alginate, wool, and polystyrene via mild and facile procedures comparing (A) previous methods of incorporation with (B) our presented work.

Mechanophores are generally introduced into synthetic polymer chains as initiators, comonomers, or cross-linkers, typically using highly controlled laboratory scale conditions, prior to polymer processing (thermoplastics) or during its formation (thermosets). In other words, mechanophores are introduced in a chemical step which is mostly unavailable in the thermoplastic industry, which typically restricts itself to thermal processes,^11^ and certainly unavailable when targeting biological tissues. When incorporated during the chemical step (say polymerization), such mechanophores still need to undergo processing, such as thermal molding in thermoplastics, which occurs under fairly aggressive conditions that can be detrimental to most mechanophores (Figure 1).^12^ Moreover, the chemical synthesis in biologically derived materials is done *in vivo* or *in vitro*, and therefore, mechanophore introduction cannot be easily accomplished prior to polymerization. If one would like to use mechanophores to introduce mechanoresponsiveness in biopolymers or tissues, an alternative strategy needs to be developed, and inspiration can be gained from other research fields like drug delivery.^13,14^ Given the requirements for near quantitative conversion and mild reaction conditions, some of the most successful chemical modifications in biomaterials are based on biorthogonal Click-chemistry.^15^ These reactions are typically done in water, under air, at room temperature, and in the absence of catalysts, making it compelling for *in situ* modification.^16^ These attributes make Click-chemistry adequate for developing a protocol of Dip-conjugation of different materials to mechanophores, which could be easily done without the complicated requirements of multistep chemical processes.

In this work, we present such a protocol, leveraging Click-conjugation of novel spiropyran (**SP**) derivatives with norbornene functionalities, to convert a wide range of materials from synthetic polymers, carbohydrates, and even proteins, including wool directly taken from an alpaca, into force-responsive materials (Figure 1). This represents a new strategy for mechanophore incorporation into bulk materials, surfaces, and networks, allowing facile and ubiquitous mechanoresponsive materials.

## Results and Discussion

### Design and Synthesis of Clickable SP

**Scheme 1:**
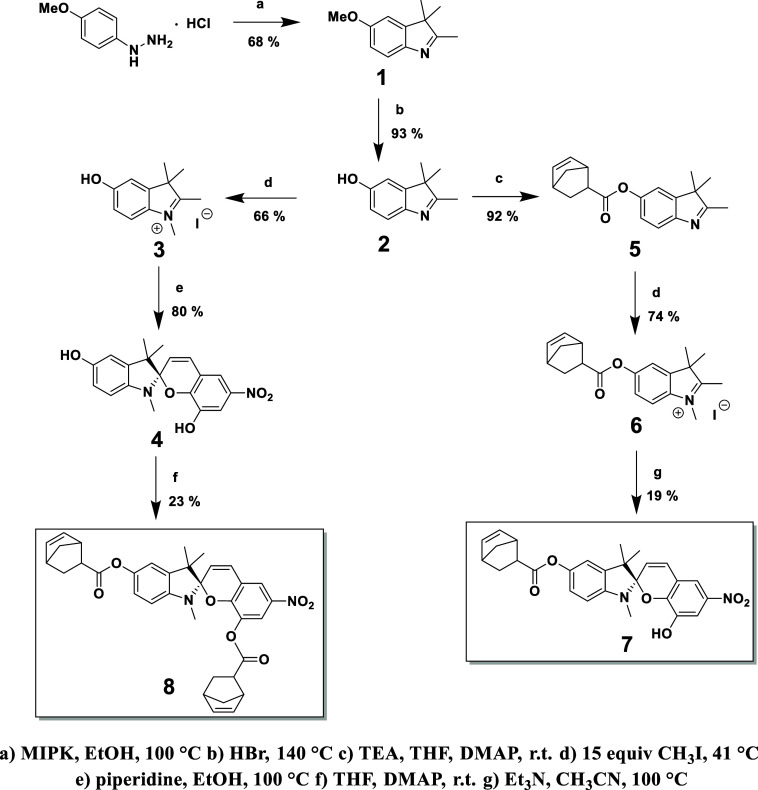
Synthesis of Compounds 7 and 8

In order to enable **SP** conjugation via Click-chemistry, we designed and synthesized two novel **SP**-norbornene derivatives, as shown in Scheme 1. As previously demonstrated, **SP** can isomerize to merocyanine with UV light irradiation, heat, low pH, or mechanical force.^17,18^ To differentiate between mechanical and other types of **SP** activation, **SP** containing a single norbornene end-group (**Compound 7**) was synthesized to make control materials. In **Compound 7**, the **SP** can be isomerized with light, heat, and changes in pH, but force propagates from the polymer only from one side and therefore does not build sufficient stress in the molecule to break the spiro C–O bond.^19^ Conversely, the new **SP** compound containing two norbornene end-groups (**Compound 8**) is sensitive to mechanical activation mechanically, as tethering both sides at the 5′ and 8’ positions translates forces to the spirojunction.^20^ Both **SP** derivative structures, after purification, were confirmed by ^1^H and ^13^C-nuclear magnetic resonance spectroscopy (NMR) and mass spectroscopy (MS) (Figures S1–S14).

To demonstrate the activity of these new **SP** derivatives connected via Click-chemistry, we decided to initially incorporate them into a rigid polymer, like polystyrene. Toward that goal, a styrene-vinyl benzyl chloride copolymer was synthesized by conventional radical polymerization and characterized by ^1^H NMR and gel-permeation chromatography (**GPC**), which indicated an average number molecular weight (***M***_***n***_) of 30 kDa (Figure S15). Then, this copolymer was substituted with azide moieties (**PS-N**_**3**_) by S_N_2 reaction with NaN_3_ (Figures S15–S17).^21,22^ Finally, we conjugated **Compound 7** and **Compound 8** to the polymer by dissolving each of them respectively (10 mg, 16.8 μM) in **PS-N**_**3**_ solution (190 mg in 10 mL DCM) and heating to 70 °C for 1 day (Figure S18),^23^ affording the mechanophore-decorated **PS-Mono** and **PS-Bis**. Conversion was determined to be quantitative as the norbornene double-bond peak at δ 6.31–5.88 ppm was not detected by ^1^H NMR in the isolated polymer (Figure S17). Given that **PS-Bis** is a cross-linked material, we prepared a control with a similar amount of a norbornene cross-linker **Compound 9** (Figure S17) and added it to **PS-Mono** and **PS-N**_**3**_ to cross-link them and reach the same overall cross-link density. According to Figure S17, the mechanophore decoration in **PS-Bis** and **PS-Mono** was estimated to be 5.14 mol% (0.52 and 0.41 wt%, respectively) based on the amount of norbornene consumed. The respective cross-linked solutions were evaporated overnight to produce thin films and die-cut into dog-bone shapes.

### Mechanoresponsiveness of Clickable SP in Polystyrene

**Figure 2:**
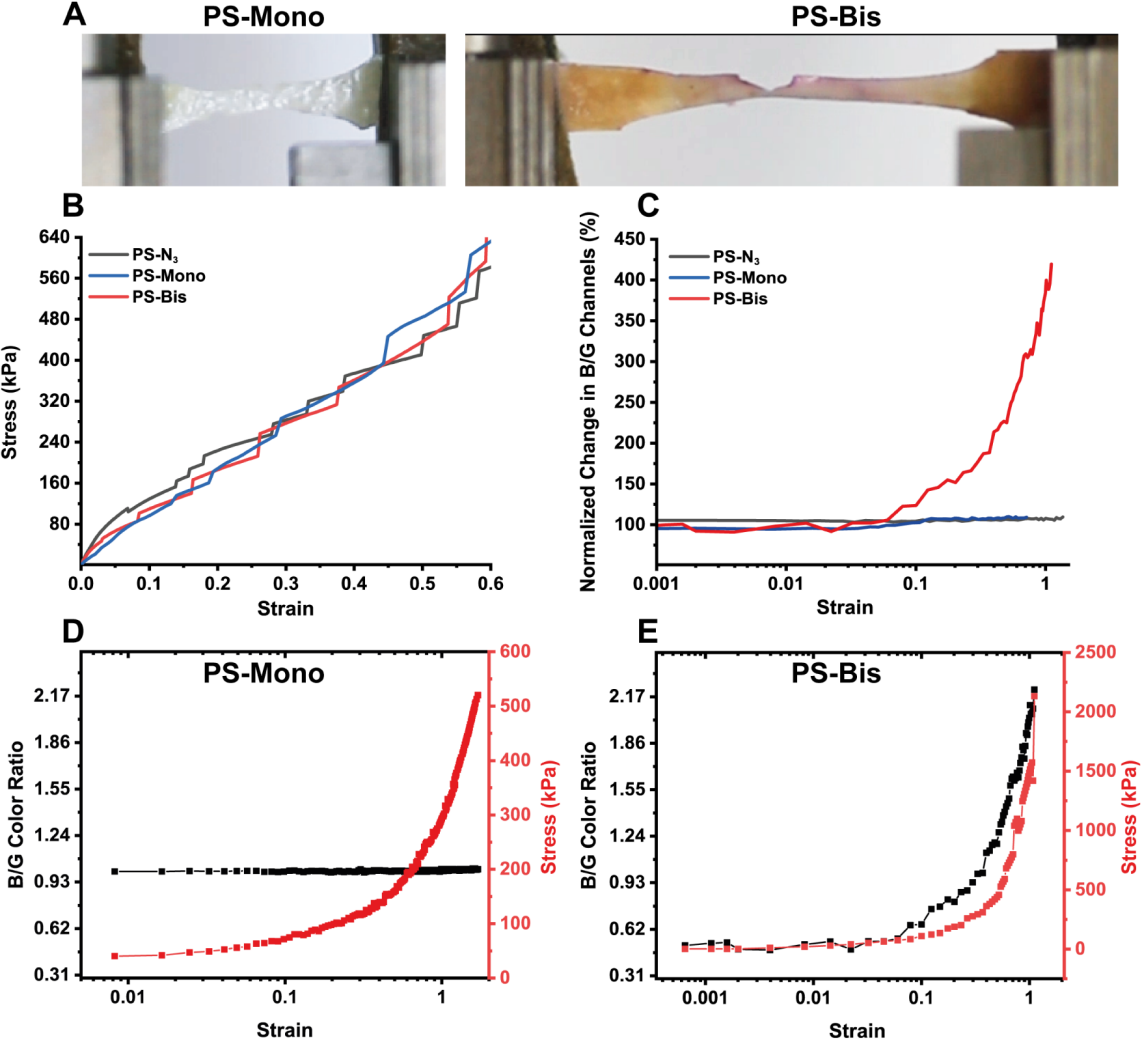
Uniaxial tensile testing via DMA coupled to video analysis shows mechanochemical activation of Dip-conjugated spiropyran. (A) Representative images of **PS-Mono** (white) and **PS-Bis** (colored) under tensile stress at the breaking point, scale bar is 0.5 cm. (B) Stress–strain plot of **PS-N**_**3**_, **PS-Mono**, and **PS-Bis** dog-bone samples. (C) Colorimetric changes in the sample purple color (Blue/Green ratio) measured along the intermediate level, normalized to 2 mm above the clamping area. Colorimetric changes and stress in dog-bone structures for (D) **PS-Mono** and (E) **PS-Bis** as functions of uniaxial strain.

**Figure 3:**
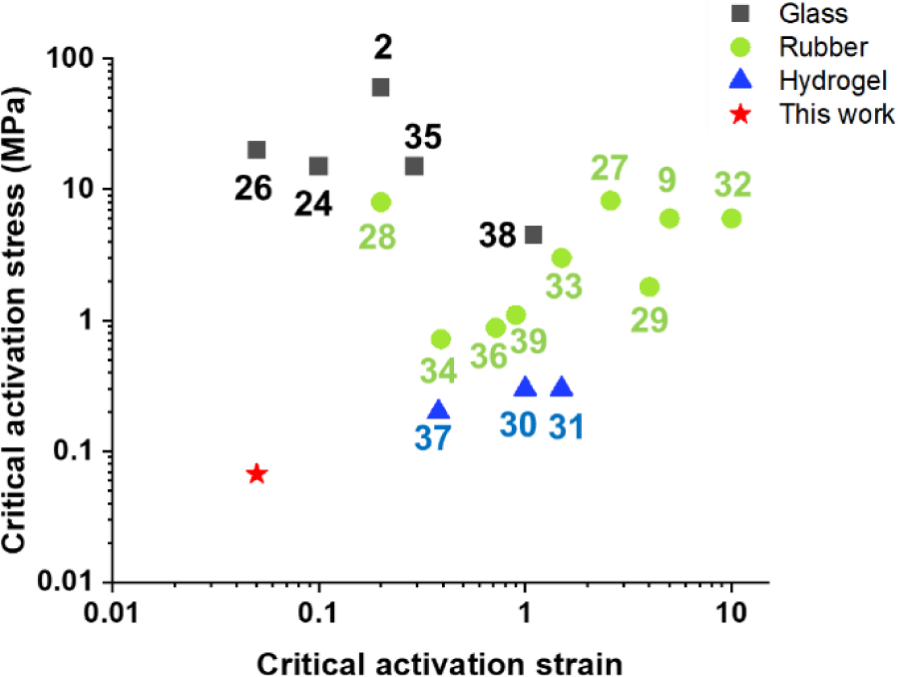
Comparison of the Spiropyran Minimum Stress and Strike Activation in the literature. Comparing the Dip-conjugation technique with the literature of spiropyran incorporation into bulk polymeric materials and their minimum stress and strain activations for glass, rubber, and hydrogel systems.^2,9,24,26−39^

While the **SP** was connected to the **PS** network in solution, to test if the Click reaction leads to a mechanoresponsive **SP** network, the sensitivity of Click-conjugated **SP** activation was characterized by using simultaneous uniaxial tensile testing and colorimetry in a dynamic mechanical analysis (**DMA**) machine. Given the high *T*_g_ of cross-linked **PS**, prior to mechanical testing, the dog bones were plasticized with 40 wt% DMF:1,4-dioxane (1:1 v/v).^24^ Each sample was loaded onto the DMA under a constant load rate of 0.2600 N/min from 0 to 2.000 N (Figure 2A). Three different samples were prepared: a “Blank” polystyrene sample made from thermoplastic **PS-N**_**3**_; **PS-Mono,** with a monofunctional conjugated **SP** (**Compound 7** and **Compound9**); and the **PS-Bis**, conjugated with the bifunctional **SP** (**Compound 8**). Uniaxial tension indicated all three samples had similar Young’s moduli (ca. 250 kPa) (Figure 2B). However, when measuring the Blue-to-Green (**B/G**) color channel ratios of the dog-bone structures, which is indicative of the conversion of **SP** to merocyanine,^19,25^ significant changes were seen in the **PS-Bis** samples only (Figure 2C). Surprisingly, this change in a signal representing mechanophore activation reflects inherent competition between energy dissipation and was already detected at 5% strain (Figures 2D and S19), which is an order of magnitude more sensitive than most other rubbery or even glassy polymers where **SP** had previously been incorporated (Figure 3).^2,9,24,26−39^ This may be due to the mechanophore activation.^40−42^ Often in bulk materials, chain scission may dominate and increase the stress on neighboring strands, which lowers the potential for force to propagate through the mechanophore.^40^ In this case where **SP** is acting as a cross-linker, the competition with premature network failure may be diminished, leading to more uniform force distribution. Only negligible B/G color ratio changes were seen in the **PS-N**_**3**_ and **PS-Mono** samples, even at strains exceeding 100%, supporting the mechanical nature of the **SP** activation in **PS-Bis**.

Though the **PS-Mono** samples were not stimulated by strain, **SP** was in fact still active following the conjugation step, as illustrated by irradiation experiments demonstrating successful photochemical **SP** activation (Figure S20). The **PS-Bis** samples were also shown to activate as a result of compression stamping by hand, a configuration that was reversible with exposure to white light (Figure S21). As the controls suggest, **SP** can be mechanically activated only when tethering both of its ends to the polymer. The main difference in our protocol is that unlike the material-specific and generally controlled conditions previously required (Table S1), this norbornene-functionalized **SP** should connect with any material having Click-capable chemistry. For comparison, we prepared a chain-centered **SP-PS-N**_**3**_ (Figures S22 and S23) and formed a thin film while cross-linking with **Compound 9**. This material was tested under tension (after plasticization as described above), and the results demonstrate that it is incapable of activation in excess of 40% strain, at which the thin film ruptures (Figure S23). By shearing the film, we were able to observe fluorescence activation at the rupture site exclusively (Figure S24). This suggests that the method of postpolymerization spiropyran incorporation, and therefore the placement within a network, may be a driver of mechanophore sensitivity.

### Mechanoresponsiveness of Clickable SP in Alginate

Given the success of this simple approach in plasticized networks, we proceeded to test the universality of our protocol with naturally derived polymers like alginate. To make alginate Click-reactive, tetrazine-decorated alginate (**ALG-Tetrazine**) was synthesized as previously described.^43^ We characterized the degree of tetrazine substitution of available carboxylic acid groups to be 2.8 mol% per monomer by UV–vis titration (Figure S25). **ALG-Tetrazine** (30 mg) was dissolved in 2 mL of deionized water (**DIW**) and a 1 mL solution of **Compound 7** or **8**, respectively (8.4 mM/mL in acetone), and was added dropwise under stirring. After 2 min, **ALG-Mono** and **ALG-Bis** were correspondingly produced (Figure S26). The degree of functionality as a result of the Click-conjugation was 2.0 mol% per monomer (4 wt%) by ^1^H NMR, based on the amount of tetrazine consumed (Figures S25 and S27). Then, thin films were prepared by drying the **ALG-Mono** and **ALG-Bis** solutions on spin-coated Teflon surfaces in the air for several days. Similar to **PS**, mechanical activation of **SP** and reactivation of the dried alginate thin films were successfully demonstrated with compression (Figure S28), but the samples were quite brittle when dry and did not lend themselves to reliable quantification of activation sensitivity.

**Figure 4:**
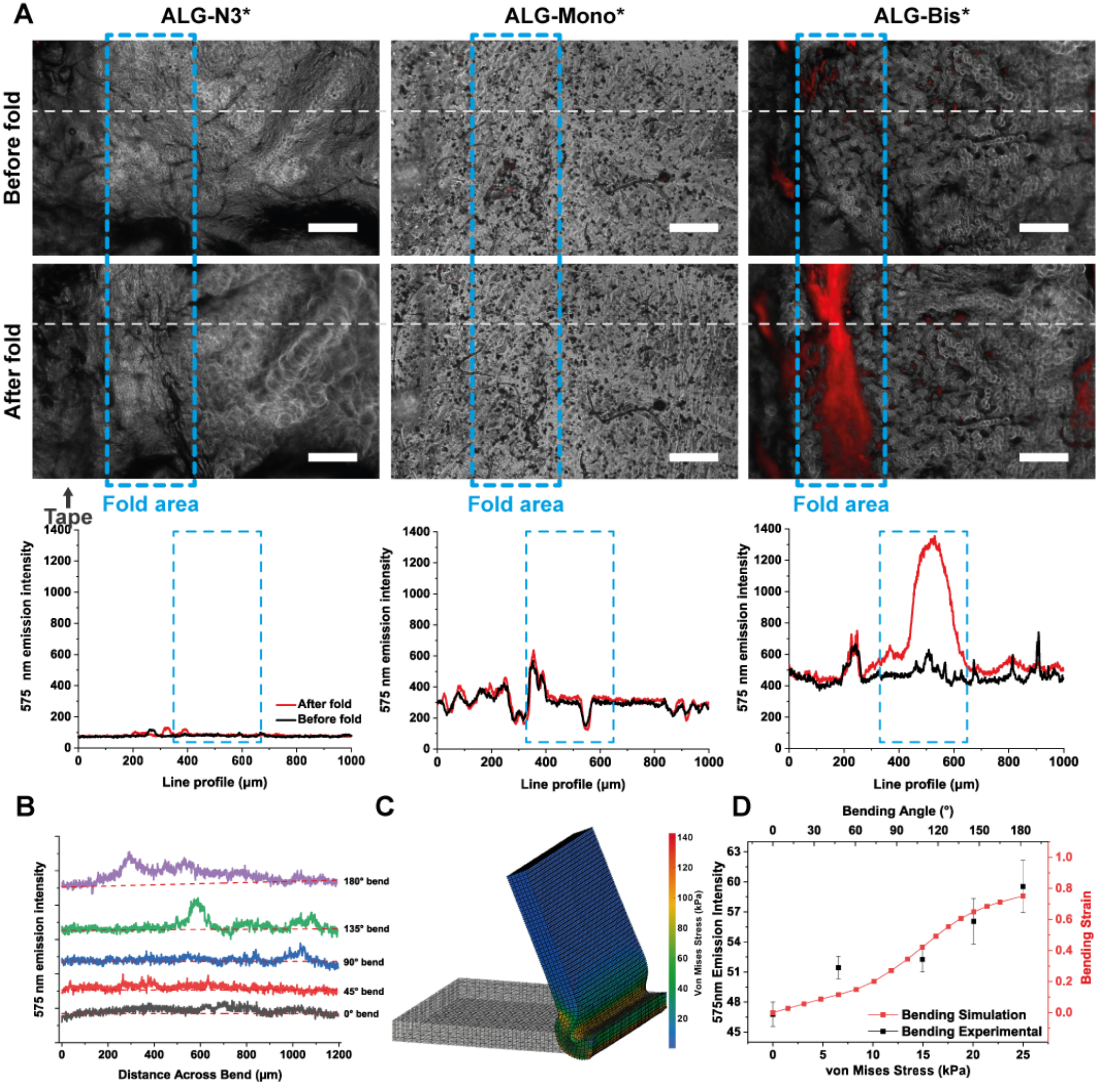
Folding of alginate thin films conjugated with spiropyran. Images were collected immediately after the fold. (A) Merged images of bright field (BF) and fluorescence emission at 575 nm with excitation at 540 nm for ALG-Tetrazine*, ALG-Mono*, and ALG-Bis* films before and after folding until 180°. The scale bar indicates 200 μm and fluorescence intensity along the dotted white line at 575 nm. (B) The fluorescence intensity of **ALG-Bis** thin films measured after bending at increasingly larger angles. (C) Representative image of the finite element analysis (FEA) model to calculate the stress distribution in an alginate thin film at 135°. (D) Correlating the bending stress and strain from FEA simulations with the experimental bending angle data across the line profile of fluorescence emissions at 575 nm of ALG-Bis*.

To enhance the durability of the brittle alginate and obtain quantifiable results, *N*,*N*-methylenebisacrylamide (6.7 × 10^–3^ molar ratio) and acrylamide (24 molar ratio) were added in accordance with prior work to form “tough alginate.”^44^ This 40 wt% water hydrogel was then subjected to folding strain, leading to successful mechanical activation of the mechanophore for the bifunctional **SP**-decorated samples (ALG-Bis*) (Figure S29), without any sample failure.^2^ As with the polystyrene model, the controls of ALG-Tetrazine* and ALG-Mono* showed only a minimal fluorescence at 575 nm (Figure 4A). Meanwhile, the ALG-Bis* samples, when folded 180°, showed a sharp increase in fluorescence at the folding site that corresponded directly with the crease formation in the film (Figure 4B). The experiment was then repeated for different folding angles (Figure S30), and it was found that the sensitivity detection limit was approximately 45° fold (Figure 4C). As with previous **SP** work, the merocyanine could be converted back to **SP** using strong white light, allowing for the reactivation of **SP** using mechanical stress. To approximate the stresses and strains associated with the folding of the mechanophore-decorated alginate films, a finite element analysis (**FEA**) model was constructed using FEBio (Figures S31–S35 and Table S3).^45^ The results of the simulated average von Mises stress calculated across the entire gel thickness at each corresponding angle (Figure 4D) were then plotted along with the experimental data for fluorescence at 575 nm integrated over the bending area (Figure 4E). Interestingly, the minimum strain required for mechanophore activation in the alginate samples was fairly similar to that found for polystyrene, at approximately 10% strain, despite the differences in mechanical test (tensile vs bending) as well as being completely different polymeric structures. The relationship between strain and color change was also remarkably linear in both cases.

### Dip-Conjugation of Clickable SP to Alpaca Wool

**Figure 5:**
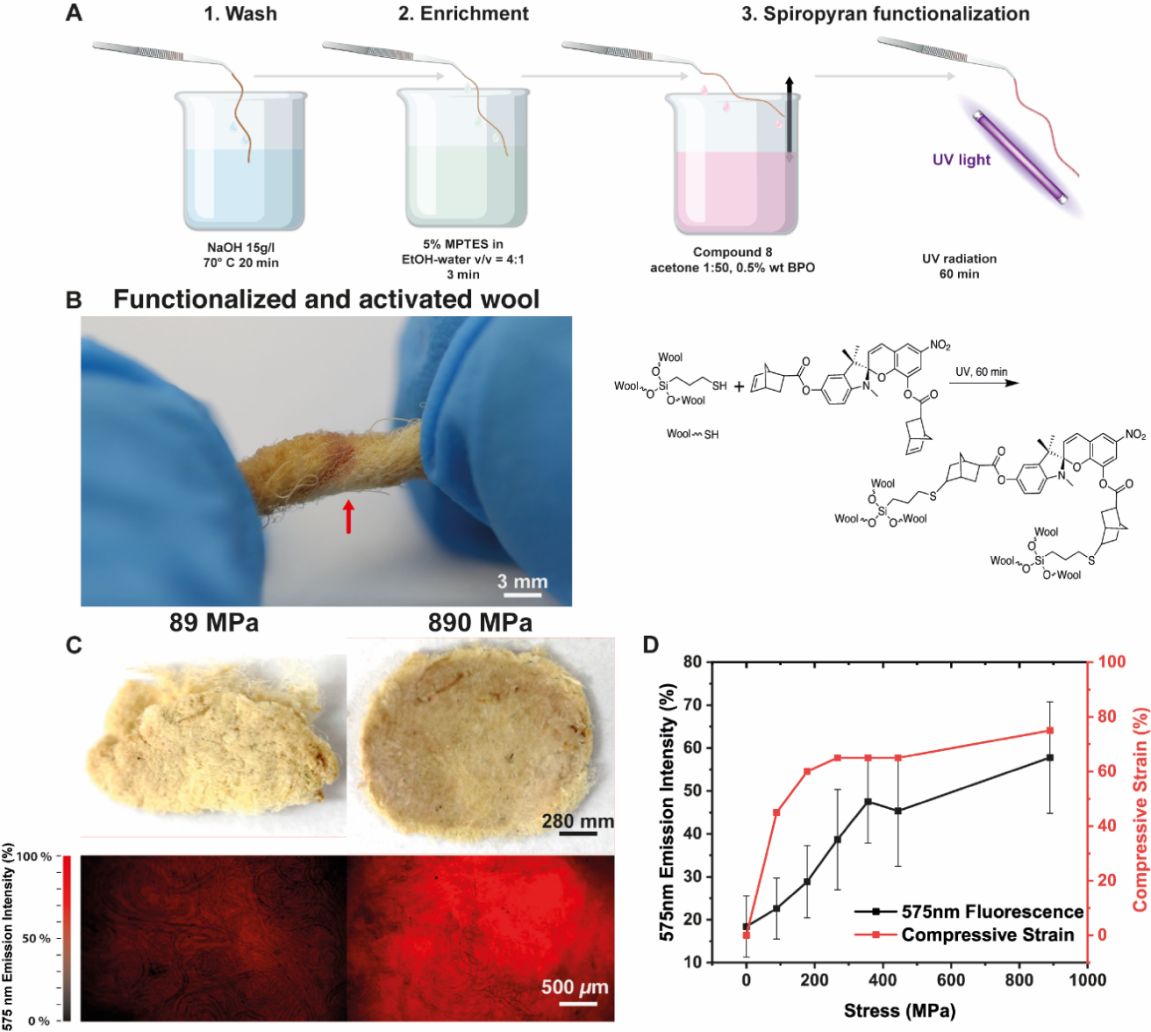
Dip-conjugating norbornene-decorated spiropyran onto alpaca wool. (A) Schematic of the protocol for Dip-conjugation and the reaction schematic of **Compound 8** with MPTES enhanced wool fibers via UV–thiolene Michael Addition (**Wool-Bis**). (B) Representative image of **Wool-Bis** and subsequent mechanical activation via twisting 180°. (C) Representative images of **Wool-Bis** compressed in a KBr pellet at 89 and 890 MPa with corresponding fluorescence at 575 nm. (D) Correlating stress and compressive strain ((*l*–*l*_0_)/*l*_0_ where *l* is the final thickness of the sample and *l*_0_ is the initial thickness) with a fluorescence intensity at 575 nm.

Next, we moved to more challenging biomaterials using naturally available functional groups on the surface of insoluble wool protein fibers, toward developing a truly universal Dip-conjugation strategy. Thiols are commonly found in many different proteins, and therefore, successful functionalization and mechanophore activation in wool opens up possibilities for a Click strategy to transform most protein-derived materials into a variety of new mechanoresponsive materials (Figure 5A). Toward this goal, we carded raw wool from alpacas and spun them into threads. Then, the wool fibers were washed with acetone for 24 h using a Soxhlet extractor to remove dirt and surface lipids (a treatment similar to that done in the wool industry). Next, the wool threads were immersed in a detergent solution of 15 g/L NaOH and heated to 70 °C for 20 min for further grease removal, making thiol and other functional groups more accessible to aqueous solutions (Figure S36). The fibers were washed with DIW to neutralize the reaction, and then, they were dried at room temperature. In order to enhance the amount of reactive thiol groups present, the fibers were immersed into a solution of 5% mercaptopropyltriethoxysilane (**MPTES**) in an ethanol-deionized aqueous solution (v/v = 4:1) for 3 min and cured under vacuum at 110 °C for 4.5 min, followed by a DIW wash.^46^ Finally, the MPTES-modified wool fibers were dipped into an acetone solution of **Compound 7** (or **8**) and BPO (photoinitiator), taken out, and irradiated under UV light (8W, 254 nm) for 1 h to maximize yields. The fibers were cleaned with DIW and dried under a vacuum to provide the final products (Figure S37).

To verify if these dip-functionalized wool fibers successfully conjugated with **Compound 8** and became mechanochromic, we initially twisted the string and found that this simple manipulation leads to a color response along the twist axis (Figure 5B). To obtain quantitative results, the wool fiber mats (**Wool-Bis**) were placed into a 13 mm diameter KBr Pellet compression mold (Shimadzu, IR spectra film preparation) and compressed at increasing pressure (between 89 and 900 MPa) for 1 min, followed by the measurement of fluorescence emission at 579 nm (Figure 5C). The data indicate an excellent correlation between the fluorescence signal strength and compressive stress exerted on the sample. As in the above examples, no color change was observed from wool conjugated with **Compound 7** (**Wool-Mono**) due to mechanical stimulation. The heterogeneity, lack of opacity, and structural organization of the wool, compared with the alginate and polystyrene samples, may bear some responsibility for the higher variability between prepared materials. That being said, the successful conjugation and activation of **SP** on naturally derived products like this is a testament to the capabilities and the potential of this strategy of Dip-conjugation.

## Conclusion

To summarize, we have designed and synthesized a novel norbornene-functionalized spiropyran, which we incorporated in different polymeric materials via Click-chemistry, first in solution and then in a novel “Dip-conjugation” protocol. To test the Click-conjugation, we incorporated the new mechanophore in a synthetic **PS** network containing azide groups and cross-linked it during casting. Next, the **SP** was conjugated to tetrazine-decorated alginate and was shown to be mechanoresponsive as well, both dry and in up to 40% water content hydrogels. Finally, we obtained alpaca wool and, after a few washing steps, functionalized it via Click-chemistry via the new “Dip-conjugation” protocol, which included a washing step, enrichment of Click-reactive functional groups, and finally Click-chemistry to the **SP** mechanophore, all without dissolving the fibers. Our results indicate that “Dip-conjugation” can be used in synthetic materials as well as those in which we have no control over polymerization chemistry, such as biologically derived materials. We demonstrated this new protocol for mechanophore incorporation into polymeric materials that is both facile, ubiquitous, and can be done postprocessing. The obtained materials showed mechanoresponsive spiropyran to merocyanine activation, providing a detailed optical history of mechanical events. Unexpectedly, this new protocol can be used with both synthetic materials in a nonchemical environment or biologically derived materials where the polymerization is out of our hands. Additionally, the process provided materials in which **SP** activation occurs as early as 5% strain, at least an order of magnitude lower compared to most previous literature reports on mechanophore activation in both glassy and rubbery bulk polymers in the solid state. This unparalleled sensitivity is general, and we observed it in glassy materials and even hydrogels. Typically, mechanophores like spiropyran require an excess of 100% strain to achieve a detectable output,^47^ well beyond linear behavior,^48^ which can lead to mechanical failure far before any detectable activation.^12^ In practical terms, this has been one of the main obstacles for even sensitive mechanophores such as spiropyran for practical commercial use and for most biological applications. It is often rare to see strains, even from growing tissues like the tumor microenvironment to exceed 10%,^49^ and for engineered plastics, larger strains fall beyond the linear regime and are of lesser value when trying to predict material failure far in advance. This technique allows us to introduce mechanophores and activate them at low strains not only in synthetic commercial polymers but, for the first time, also in materials derived from carbohydrates and proteins. This represents a breakthrough in the field of mechanochemistry and mechanobiology, particularly as interests shift to measuring forces at the chemical level in biologically derived materials and biological processes. The enhanced sensitivity of activation and ease of *in situ* conjugation put small molecule mechanophores in the range of many biological processes. It can also be applied to engineered plastics where mechanophore incorporation was previously too costly and difficult to implement with current thermoplastic molding techniques.

## Supporting Information Available

Experimental procedures, synthesis of small molecules and polymers, finite element modeling, UV–vis, controls, additional GPC traces, mass-spectroscopy, and NMR are included in the Supporting Information (PDF)
