# Supplementary material for: Facile Mechanophore Integration in Heterogeneous Biologically Derived Materials via “Dip-Conjugation”
Source: J Am Chem Soc. 2024 Jun 20;146(26):17878–86. doi: 10.1021/jacs.4c03534 (PMC11229001; doi:10.1021/jacs.4c03534)
Supplement: Supplementary file 1 — ja4c03534_si_001.pdf [file ja4c03534_si_001.pdf]

SUPPORTING INFORMATION

Facile mechanophore integration in heterogenous biologically-derived materials via "Dip-Conjugation"

Yifan Liao,[1] Baptiste Le Roi,[1] Hang Zhang,[2] Charles E. Diesendruck,[2] and Joshua M. Grolman[1*]

[1]Materials Science and Engineering Department, Technion-Israel Institute of Technology, Technion City, Haifa 3200003, Israel

[2]Shulich Faculty of Chemistry, Technion-Israel Institute of Technology, Technion City, Haifa 3200003, Israel

Table of Contents

1. Supplemental General remarks 3
2. Supplemental Experimental procedures 5
   2.1 Mechanophore Synthesis 5
   2.2 Mechanophore Characterizations 8
3. Mechanophore application in Poly(styrene) 15
   3.1 Synthesis and Characterizations 15
   3.2 Application 21
   3.3 Comparison current method and our strategy 22
4. Mechanophore application in Alginate 22
   4.1 Synthesis and Characterizations 22
   4.2 Application 25
   4.3 FE modeling 27
      4.3.1 Context 27
      4.3.2 Geometry 27
      4.3.3 Boundary, contact and loading conditions 27
      4.4.4 Computational simulations and outcome measures 28
5. Mechanophore application in Wool 31
   5.1 Synthesis and Characterizations 31
6. Supplemental References 34

1. Supplemental General remarks

All reagents for synthesis, unless otherwise noted, were purchased from Sigma-Aldrich and used as received. The synthesis of **Compound 7** ((±)-8-hydroxy-1',3',3'-trimethyl-6-nitrospiro[chromene2,2'-indolin]-5'-yl bicyclo[2.2.1]hept-5-ene-2-carboxylate), **Compound 8** ((±)-8-hydroxy-1',3',3'trimethyl-6-nitrospiro[chromene-2,2'-indolin]-5'-yl bicyclo[2.2.1]hept-5-ene-2-carboxylate) and **Compound 9** (nonane-1,9-diyl bis (bicyclo[2.2.1]hept-5-ene-2-carboxylate)) involved several chemicals obtained from different suppliers. Methyl isopropyl ketone (**MIPK**), triethanolamine (**TEA**), ethyl acetate, $NaHCO_3$, NaCl, Na_2SO_4, dichloromethane **(DCM)**, ethylether, acetone, acetonitrile (**CH_3CN**), hexane, 1-Ethyl-3-(3-dimethylaminopropyl) carbodiimide (**EDCl·HCl**) were used. Ethanol (**EtOH**), methanol (**MeOH**), and tetrahydrofuran (**THF**) were purchased from Bio-lab. 5-Norbornene-2-carboxylic acid, a mixture of 8:2 endo and exo isomers (**Fig. S1**), hydrobromic acid (**HBr**, Fisher Chemical), 4-dimethylaminopyridine (**DMAP**, Aaron Chemical), iodomethane (**CH_3I**, Acros Organics), triethylamine (**Et_3N**, Alfa Aesar) were used. For the synthesis of styrene-vinyl benzyl chloride copolymer (**PS-Cl**), 4-vinylbenzyl chloride (**VBC**), and styrene (**St**, Alfa Aesar), monomers were purified before use by passing them through basic aluminum oxide (Acros Organics) to remove stabilizing agents. Benzyl azide was prepared following procedures found in the literature.[1] CuCl was washed with acetic acid and dried under vacuum prior to use. KOH (Bio-Lab) and benzoyl peroxide (**BPO**, Merck) were used are received. For the preparation of styrene-vinyl benzyl chloride copolymer functionalized with azide (**$PS-N_3$**), sodium azide, and N, N-Dimethylformamide (**DMF**, Bio-Lab) were used. For the preparation of the spiropyran alginate, sodium alginate (180 kDa, KIMICA Corporation, Japan), and 2-(Nmorpholino) ethanesulfonic acid (**MES**, Glentham Life Sciences) were used. Dialysis tube (12-14 kDa MWCO, D9527), hydroxylamine, 1-ethyl-3-(3-dimethylaminopropyl)-carbodiimide hydrochloride (**EDC**), N-hydroxysuccinimide (**NHS**), (4-(1,2,4,5-Tetrazin-3-yl) phenyl) methanamine hydrochloride (**Tetrazine**) were used. For the preparation of the spiropyran alpaca wool (alpaca farm, Israel), NaOH (Bio-Lab), (3-Mercaptopropyl) methyldimethoxysilane (**MPTES**), 2-Hydroxy-2-methylpropiophenone, 5,5-dithio-bis-(2-nitrobenzoic acid) (Holland Moran) were used. The water used throughout all the experiments was purified from a Wasserlab water purification system (Automatic Plus Type II, Barcelona, Spain).

An AVANCE II 400 MHz (Bruker) NMR spectrometer at the Technion NMR facilities was used to record the ^1H nuclear magnetic resonance (NMR), ^{13}C NMR. All the NMR experiments were carried out at 25 °C and processed with MestReNova software. Chemical shifts (δ) for ^1H NMR spectra were referenced to protons on the residual solvent (7.26 ppm for $CDCl_3$-d_1, 4.79 ppm for D_2O-d_2). Chemical shifts (δ) for ^{13}C NMR spectra were referenced to the deuterated solvent itself (77.16 ppm for $CDCl_3$-d_1, 39.52 ppm for DMSO-d_6). NMR-spectroscopic data are reported as follows: chemical shift (δ in ppm), multiplicity (s=singlet, d=doublet, t=triplet, m=multiplet, dd=doubledoublet, td=triplet-doublet, tdd=triplet-doublet-doublet, ddd=double-double-doublet, dddd=doubledouble-doublet-doublet), coupling constants (J in Hz) and integration.

High - resolution mass spectrometry was performed on a Waters LCT Premier Mass Spectrometer (ESI) and a Bruker maxis impact with an APCI solid probe.

Silica gel column chromatography was performed using silica gel (particle size 40–64 μm, 230–400 mesh).

Gel permeation chromatography (GPC) analyses were carried out using a Thermo LC system. According to the polymer's solubility, the system was eluted with THF, at 30 °C at 1 mL/min and calibrated using monodisperse poly(styrene) standards. The THF system consisted of one Tosoh TSKgel HHR-L guard column and four TSKgel G4000HHR columns in sequence. The Relative scale is refractive index (RI) from ultraviolet detector. And the M_w is calculated by Mark-Houwink-sakurada K with 1.000 mL/g.

UV-Vis measurements were taken using a Thermo Evolution 220 spectrophotometer after diluting the sample in a solvent.

The UV photoreaction setup consisted of 2 UV lamps (Philips TUV 8W/G8T5) with a power rating of 8 W and a dominant wavelength of 254 nm.

pH readings were obtained using an AZ 86505 pH/mV/Cond./TDS/Temperature meter with SIN:1104961. The digital images and video were obtained using a Canon DS126291 camera with a Canon Macro Lens EF-S 60 mm 1: 2.8 USM lens under ambient room light conditions.

Attenuated total reflection-Fourier transform infrared (ATR-FTIR) spectra were recorded on a Thermo Scientific Nicolet FTIR iS50 spectrometer at room temperature in the 400-4000 cm^{-1} range. Powder samples pelleted with anhydrous 200 mg KBr (99+%, FTIR grade, Sigma-Aldrich) were used and compared with freshly prepared blank KBr pellets.

Images of fluorescence variation of the mechanochromic material were acquired on a fluorescence microscope (Nikon ECLIPSE Ts2R, Japan) using a 4x phase contrast objective (CFI Super Plan Fluor ELWD ADM, China) equipped with a high-resolution digital camera from Hamamatsu (ORCASpark Digital CMOS camera C11440-36U). The fluorescence image was acquired with a laser excitation of 460 nm, and the emission was observed with a 575 nm RFP filter. All the microscope images were processed and analyzed using Fiji (https://imagej.net/Fiji) or NIS-Elements AR 5.41.01 software (https://www.microscope.healthcare.nikon.com).

Tensile tests were conducted using a dynamic mechanical analyzer (Q800 DMA, TA Instruments) with a constant strain load rate of 0.2600 N/min to 2.0000 N. The samples were cut into dog-bone shapes. The specimens were exposed to white light for approximately 5 min before the test to erase the purple color along the edges caused by the force-induced SP-MC transition.

References 2 and 26 of data in **Figure 3** were acquired using the Import Data capabilities of Origin2021b and the critical strain was obtained by taking the first non-zero derivative of the colorimetric response curve. Other values were reported from the cited references.

2. Supplemental Experimental procedures

2.1 Mechanophore Synthesis

Compounds **1-4** (**Scheme 1**) and norbornene anhydride are known products.[2-5]

Synthesis of 2,3,3-trimethyl-3H-indol-5-yl bicyclo[2.2.1]hept-5-ene-2-carboxylate (5)

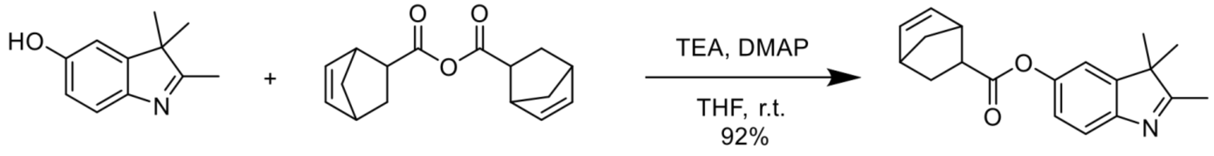

2 **Norbornene Anhydride** **5**

Scheme S1: Synthesis of 2,3,3-trimethyl-3H-indol-5-yl bicyclo[2.2.1]hept-5-ene-2-carboxylate

Compound **2** (600 mg, 3.42 mmol, 1.00 equiv) and TEA (0.50 mL, 3.59 mmol, 1.05 equiv) were dissolved in THF (31 mL). The mixture was dropwise added with norbornene anhydride (1.23 g, 4.77 mmol, 1.40 equiv) and DMAP (62 mg, 0.51 mmol, 0.15 equiv) in THF (5 mL) at 0°C. After the mixture was stirred at room temperature overnight, the reaction was quenched with MeOH (1 mL) and diluted with ethyl acetate. The organic phase was washed with a saturated $NaHCO_3$ solution and NaCl several times then dried with anhydrous Na_2SO_4. After filtration, the product was purified by silica gel column chromatography using 30:1 DCM/MeOH as eluent. The final purified product **5** was obtained as a yellow viscous liquid. Yield: 460 mg (92%); ^{1}H NMR (400 MHz, $CDCl_3$-d_1): δ 7.49 (t, *J* = 8.3 Hz, 1H), 7.04-6.98 (m, 1H), 6.97 – 6.90 (m, 1H), 6.30-6.04 (m, 2H), 3.45-3.28 (s, 0.44H), 3.27-3.16 (m, 1H), 3.05-2.92 (m, 1H), 2.55-2.42 (m, 0.45H), 2.26 (d, *J* = 3.2 Hz, 3H), 2.11-2.05 (m, 0.45H), 2.03-1.97 (m, 0.54H, H_1-exo), 1.58-1.33 (m, 3H), 1.30 (d, *J* = 4.9 Hz, 6H);^{13}C NMR (101 MHz, $CDCl_3$-d_1): 188.05, 174.85, 150.85, 148.51, 146.64, 138.12, 135.47, 131.99, 119.93, 114.97, 53.86, 49.59, 46.64, 46.24, 41.58, 30.42, 22.84, 15.24.

Synthesis of 5-((bicyclo[2.2.1]hept-5-ene-2-carbonyl)oxy)-1,2,3,3-tetramethyl-3H-indolium iodide (6)

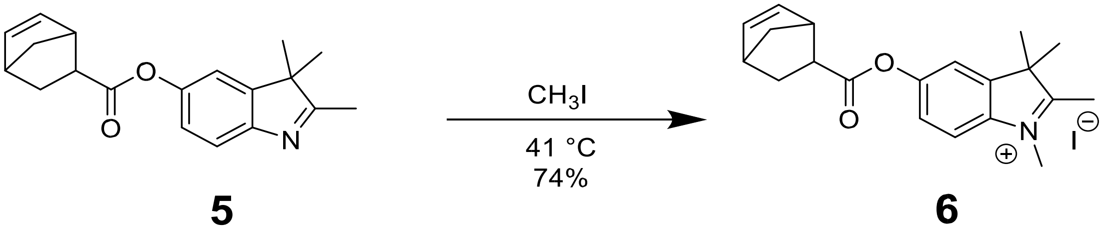

5 **6**

Scheme S2: Synthesis of 5-((bicyclo[2.2.1]hept-5-ene-2-carbonyl)oxy)-1,2,3,3-tetramethyl-3Hindolium iodide

A solution of compound **5** (136 mg, 0.46 mmol, 1.00 equiv) in CH_3I (0.44 mL, 6.90 mmol, 15.0 equiv) was stirred at 41 °C for 24 h. The mixture was filtered and washed with cold ethyl ether. The residue was dissolved in a small amount of acetone and then dropwise added to a cold rapidly stirred ethyl ether solution resulting precipitate. Product **6** was obtained by filtration as a white powder. Yield: 149 mg (74%); ^{1}H NMR (400 MHz, $CDCl_3$-d_1): δ 7.76 (t, *J* = 8.6 Hz, 1H), 7.33-7.26 (m, 1H), 7.25-7.19 (m, 1H),

6.29-6.02 (m, 2H), 4.20 (dd, *J* = 3.7, 1.0 Hz, 3H), 3.37 (s, 0.43H), 3.293.16 (m, 1H), 3.04 (dd, *J* = 5.1, 1.0 Hz, 3H), 2.99 (s, *J* = 3.5 Hz, 1H), 2.49 (ddd, *J* = 9.0, 4.4, 1.4 Hz, 0.54H), 2.02 (dddd, *J* = 11.7, 7.1, 3.7, 2.5 Hz, 1H), 1.71 (s, 0.88H), 1.62 (d, *J* = 4.5 Hz, 6H), 1.58-1.42 (m, 3H);^{13}C NMR (101 MHz, DMSO-d_6): 196.62, 174.63, 173.18, 151.78, 143.61, 140.06, 138.63, 136.02, 132.85, 123.02, 118.04, 116.63, 54.55, 46.53, 43.08, 41.67, 35.48, 30.71, 22.11, 14.84.

Synthesis of (±)-8-hydroxy-1',3',3'-trimethyl-6-nitrospiro[chromene-2,2'-indolin]-5'-yl bicyclo[2.2.1]hept-5-ene-2-carboxylate (7)

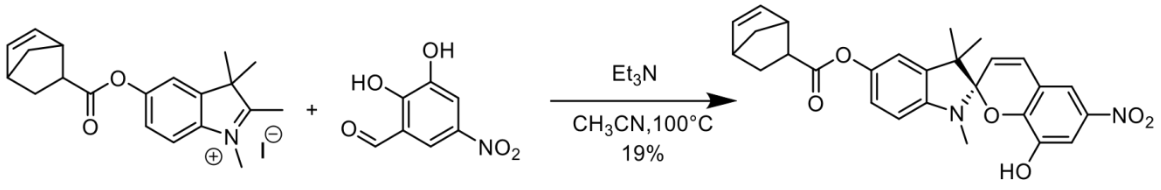

6 **7**

Scheme S3: Synthesis of (±)-8-hydroxy-1',3',3'-trimethyl-6-nitrospiro[chromene-2,2'-indolin]-5'-yl bicyclo[2.2.1]hept-5-ene-2-carboxylate

The compound **6** (437 mg, 1.00 mmol, 1 equiv), 2,3-dihydroxy-5-nitrobenzaldehyde (183 mg 1.00 mmol, 1 equiv), and Et_3N (0.28 mL, 2.00 mmol, 2 equiv) were dissolved in CH_3CN (9.5 mL) and reflux at 100 °C for 5 h. Then the product was purified by silica gel column chromatography using 50:1 DCM/MeOH as eluent to obtain **7** as a black solid. Yield: 90.1 mg (19%); ^{1}H NMR (400 MHz, $CDCl_3$-d$_1$): δ 7.69 (ddd, *J* = 18.8, 2.6, 1.7 Hz, 2H), 7.06-6.67 (m, 3H), 6.53 (t, *J* = 8.5 Hz, 1H), 6.37-6.05 (m, 2H), 5.92-5.76 (m, 1H), 3.41 (s, 0.48H), 3.29-3.17 (m, 1H), 3.02 (s, 1H), 2.76 (d, *J* =4.6 Hz, 3H), 2.50 (ddd, *J* = 8.9, 4.5, 1.6 Hz, 0.54 H), 2.13-1.99 (m, 1H), 1.82 (d, J = 5.3 Hz, 0.79H), 1.64 (d, *J* = 8.5 Hz, 1H), 1.51-1.36 (m, 1.39H), 1.26 (dd, *J* = 25.4, 4.7 Hz, 6H); ^{13}C NMR (101 MHz, $CDCl_3$-d_1): 173.64, 172.20, 151.33, 145.07, 140.04, 138.18, 135.75, 132.23, 128.40, 119.91, 119.41, 115.44, 107.29, 52.02, 49.80, 49.40, 46.00, 45.29, 43.61, 42.73, 41.79, 31.63, 29.44, 28.89, 22.69, 19.59, 14.17; HRMS (ESI+) *m/z*: [M + H$^+$] calcd 475.1869; found 475.1876.

Synthesis of (±)-1',3',3'-trimethyl-6-nitrospiro[chromene-2,2'-indoline]-5',8-diyl bis(bicyclo[2.2.1]hept-5-ene-2-carboxylate) (8)

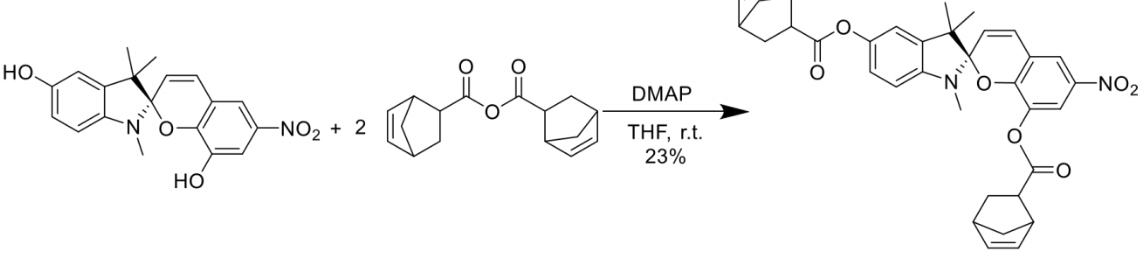

4 **8**

Scheme S4: Synthesis of (±)-1',3',3'-trimethyl-6-nitrospiro[chromene-2,2'-indoline]-5',8-diyl bis(bicyclo[2.2.1]hept-5-ene-2-carboxylate)

4 (300 mg, 0.84 mmol, 1.0 equiv) and DMAP (187 mg, 1.52 mmol, 1.8 equiv) were fully dissolved into THF (7.5 mL), followed by the dropwise addition of the norbornene anhydride (564 mg, 2.18 mmol, 2.6 equiv). After stirring for 7 h at room temperature, the reaction was quenched with MeOH (1 mL). The crude product was purified by column chromatography eluting with 50:1 DCM/MeOH. After removing the solvent, the product was purified by recrystallization from boiling hexane to afford **8** as a shallow green powder. Yield: 124 mg (23%); ^{1}H NMR (400 MHz, $CDCl_3$-d_1): δ 8.07-7.63 (m, 2H), 7.13-6.67 (m, 3H), 6.47 (td, *J* = 8.5, 4.8 Hz, 1H), 6.31-5.93 (m, 3.38H), 5.88 (ddd, *J* = 10.3, 4.8, 2.2 Hz, 1H), 5.62 (ddd, *J* = 41.1, 5.8, 3.0 Hz, 0.6H), 3.45-2.70 (m, 3H), 2.542.39 (m, 0.4H), 2.17 (s, 0.4H), 2.11-1.94 (m, 1H), 1.88-1.58 (m, 1H), 1.53-1.27 (m, 5H), 1.23-1.09 (m, 6H), 1.08-0.94 (m, 0.76H); ^{13}C NMR (101 MHz, $CDCl_3$-d_1): 173.62, 172.19, 151.31, 145.06, 140.03, 138.16, 137.42, 135.73, 132.22, 128.38, 121.30, 120.05, 119.40, 115.42, 107.54, 107.27, 52.00, 49.78, 49.39, 46.84, 46.42, 45.98, 45.27, 43.60, 42.71, 42.33, 41.78, 31.61, 30.61, 29.42, 28.87, 25.81, 22.68, 19.57, 14.15; HRMS (ESI+) *m/z*: [M + H^+] calcd 595.2439; found 595.2439.

Synthesis of nonane-1,9-diyl bis(bicyclo[2.2.1]hept-5-ene-2-carboxylate) (endo/exo = 3/1)(9)

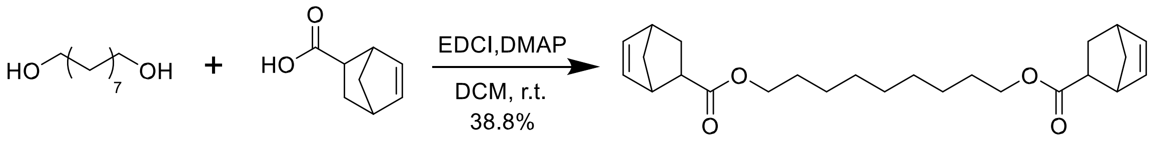

9

Scheme S5: Synthesis of nonane-1,9-diyl bis(bicyclo[2.2.1]hept-5-ene-2-carboxylate) (endo/exo = 3/1). In a Schlenk flask, Nonane-1,9-diol (1 g, 6.24 mmol, 1.0 equiv), norbornene acid (3.45 g, 24.96 mmol, 4.0 equiv) and DMAP (0.3g, 2.5 mmol, 0.4 equiv) were dissolved in 120 mL of anhydrous DCM. Then EDCI·HCl (4.8 g, 24.96 mmol, 4.0 equiv) was added in several portions into the mixture which was cooled down to 0 °C. Then, the reaction was left to stir at R.T. for 24 h. The crude product was purified by column chromatography eluting with 3:1 Hexane/DCM. Upon solvent evaporation, the product was afforded as a transparent solution. Yield: 970 mg (38.8%); ^{1}H NMR (400 MHz, $CDCl_3$-d_1): δ 6.21-5.87 (m, 4H), 4.15-3.88 (m, 4H), 3.23-2.87 (m, 5.35H), 2.252.17 (dd, J = 10.3, 4.4 Hz, 0.46H), 1.95-1.85 (tdd, J = 12.1, 7.9, 3.9 Hz, 2H), 1.67-1.49 (m, 5.45H), 1.45-1.40 (m, 2.86H), 1.39-1.23 (m, 12H); ^{13}C NMR (101 MHz, $CDCl_3$-d_1): 176.35, 174.86, 138.05, 137.75, 135.80, 132.38, 64.57, 64.32, 49.64, 46.64, 46.38, 45.74, 43.38, 43.23, 42.55, 41.65, 30.33, 29.41, 29.39, 29.19, 29.16, 28.70, 28.68, 25.94; HRMS (ESI+) *m/z*: [M + H^+] calcd 401.2686; found 401.2689.

2.2 Mechanophore Characterizations

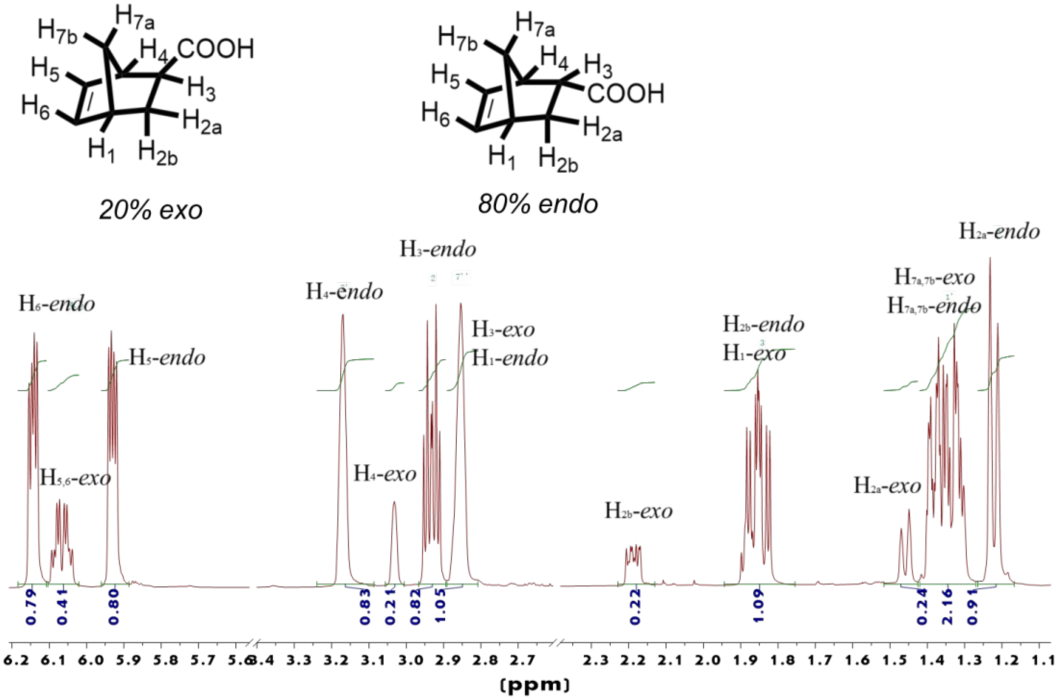

Figure S1: ^{1}H NMR (400 MHz, $CDCl_3$-d_1) spectra of the starting material norbornene acid 20% exo and 80% endo monomers.

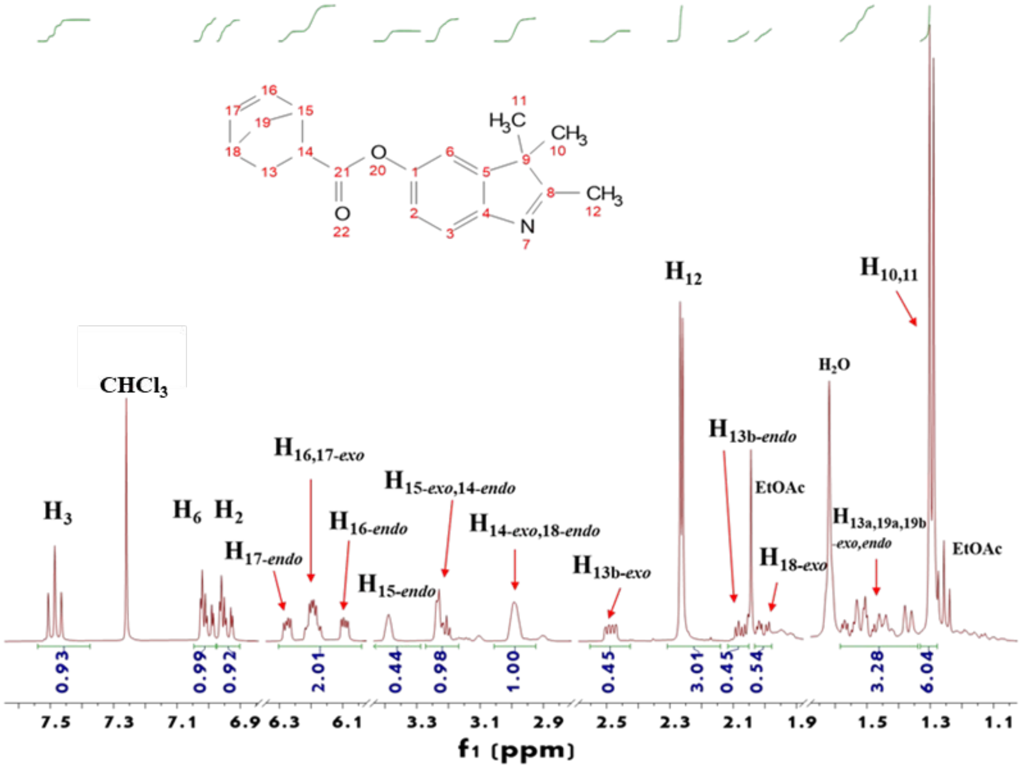

Figure S2: ^{1}H NMR spectra (400 MHz, $CDCl_3$-d_1) of 2,3,3-trimethyl-3H-indol-5-yl bicyclo[2.2.1]hept-5-ene-2-carboxylate (**5**).

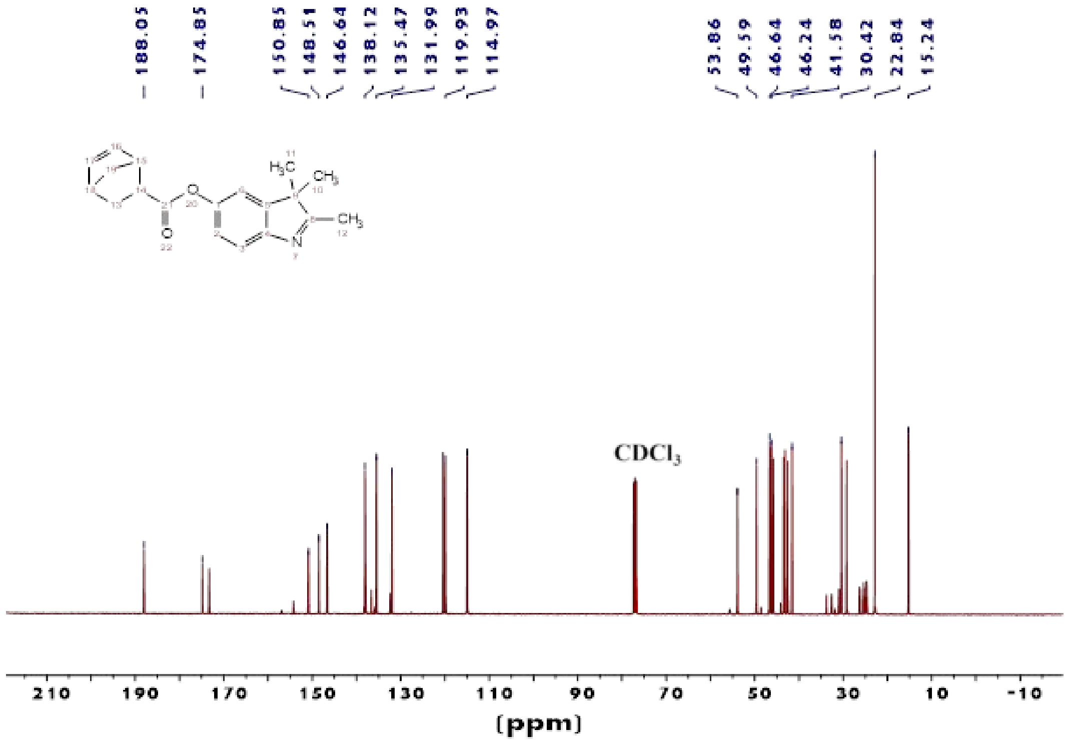

Figure S3: ^{13}C NMR spectra (101 MHz, CCl_3-d_1) of 2,3,3-trimethyl-3H-indol-5-yl bicyclo[2.2.1]hept5-ene-2-carboxylate (**5**).

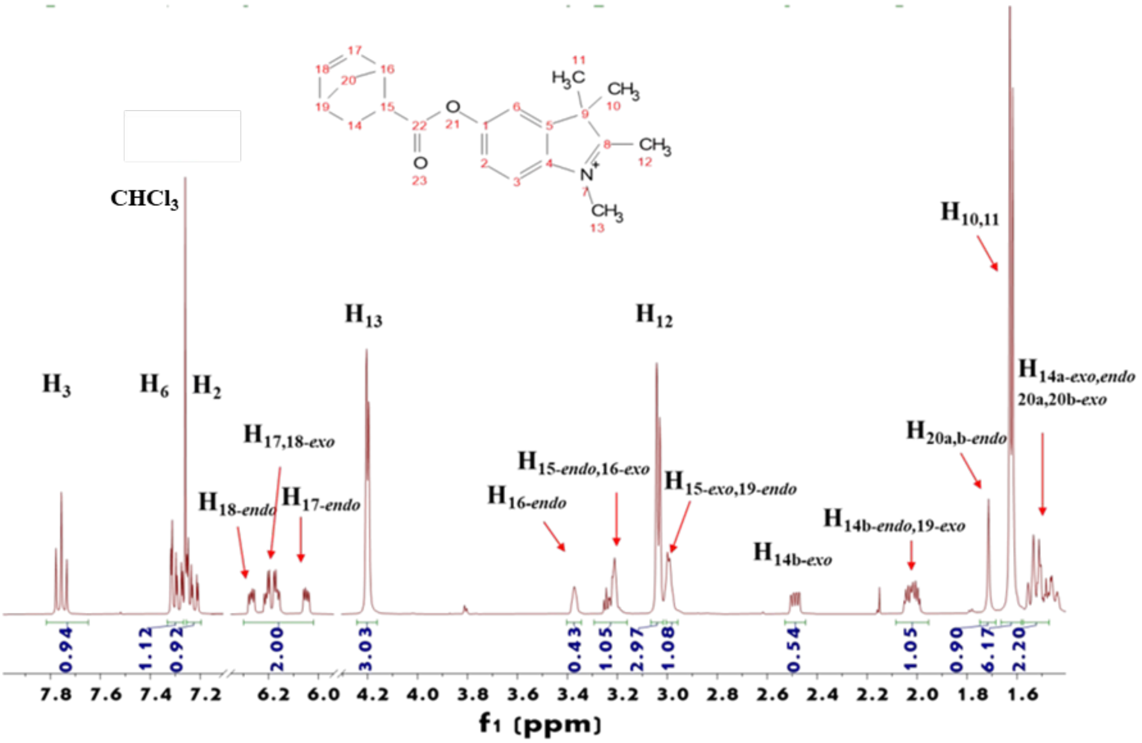

Figure S4: ^{1}H NMR spectra (400 MHz, $CDCl_3$-d_1) of 5-((bicyclo[2.2.1]hept-5-ene-2-carbonyl)oxy)-1,2,3,3-tetramethyl-3H-indolium iodide (**6**).

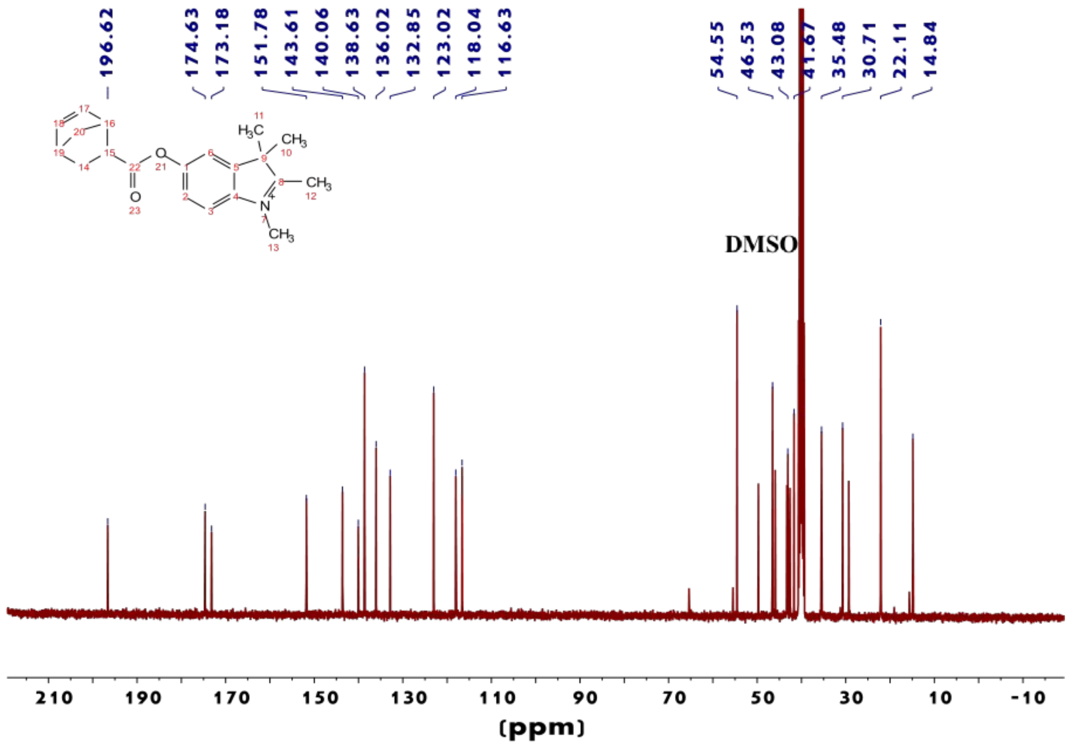

Figure S5: ^{13}C NMR spectra (101 MHz, DMSO-d_6) of 5-((bicyclo[2.2.1]hept-5-ene-2-carbonyl)oxy)-1,2,3,3-tetramethyl-3H-indolium iodide (**6**).

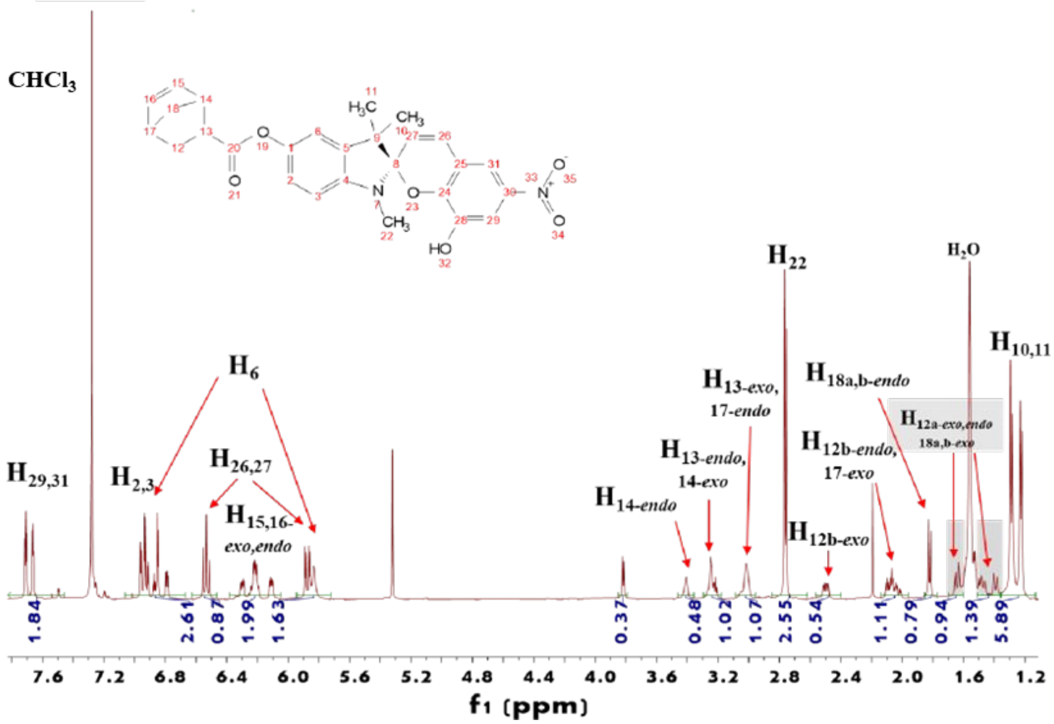

Figure S6: ^{1}H NMR spectra (400 MHz, CDCl$_3$-d_1) of (±)-8-hydroxy-1',3',3'-trimethyl-6nitrospiro[chromene-2,2'-indolin]-5'-yl bicyclo[2.2.1]hept-5-ene-2-carboxylate (**7**).

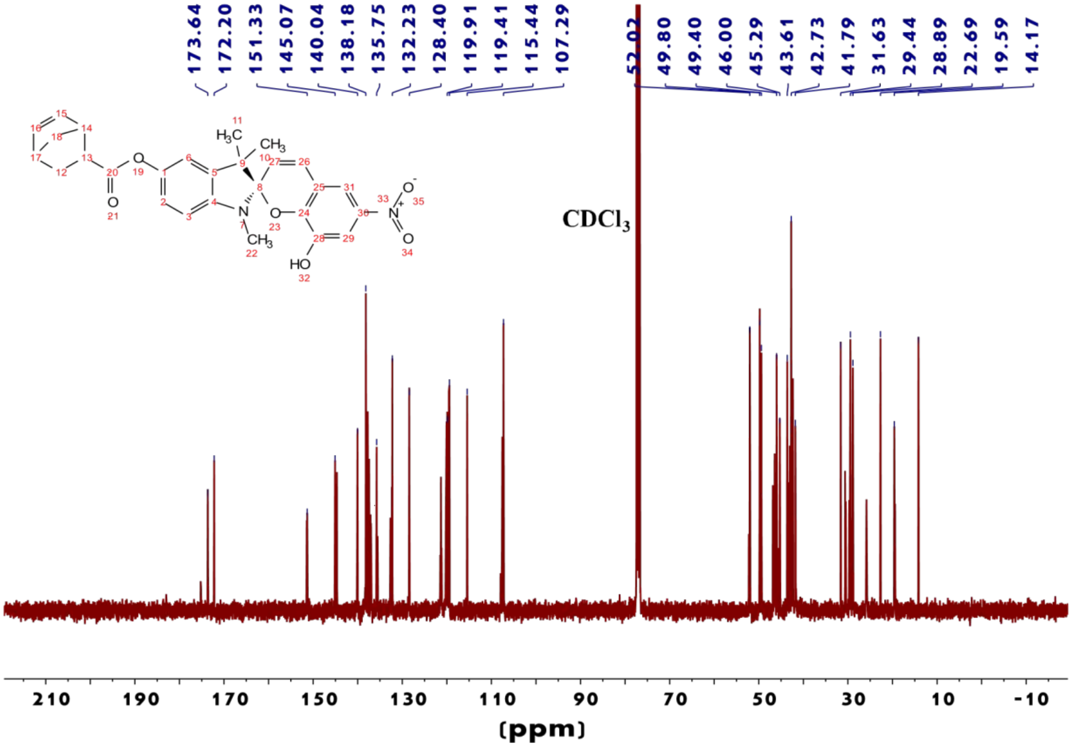

Figure S7: ^{13}C NMR spectra (101 MHz, DMSO-d_6) of (±)-8-hydroxy-1',3',3'-trimethyl-6-nitrospiro[chromene-2,2'-indolin]-5'-yl bicyclo[2.2.1]hept-5-ene-2-carboxylate (**7**)

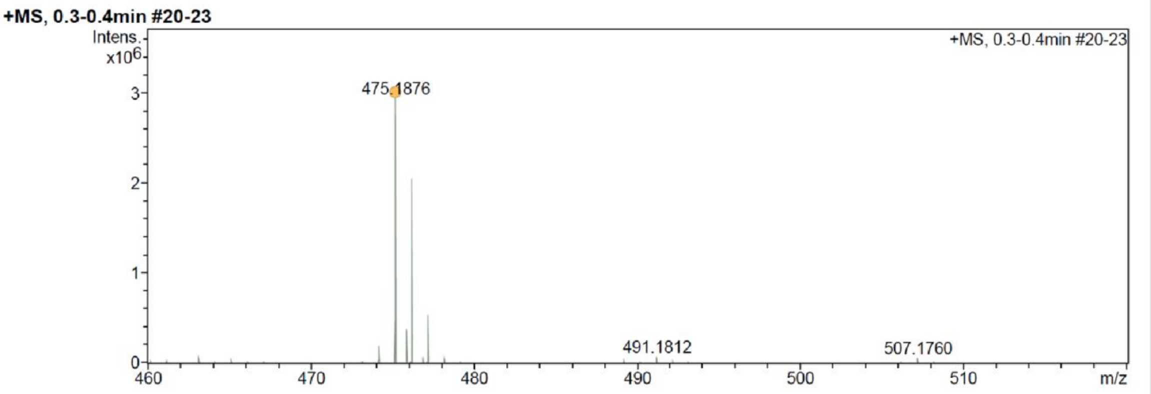

Figure S8: Mass spectra of (±)-8-hydroxy-1',3',3'-trimethyl-6-nitrospiro[chromene-2,2'-indolin]-5'-yl bicyclo[2.2.1]hept-5-ene-2-carboxylate (**7**) were obtained using an APCI source type.

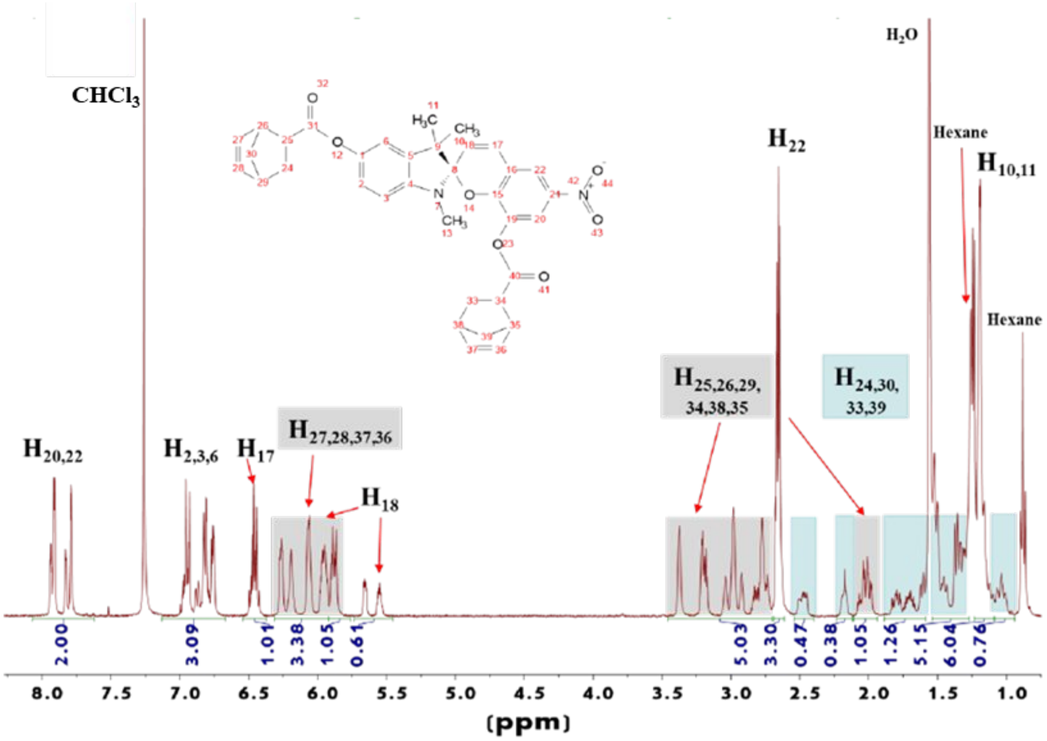

Figure S9: ^{1}H NMR spectra (400 MHz, $CDCl_3$-d_1) of (±)-1',3',3'-trimethyl-6-nitrospiro[chromene2,2'-indoline]-5',8-diyl bis(bicyclo[2.2.1]hept-5-ene-2-carboxylate)(**8**).

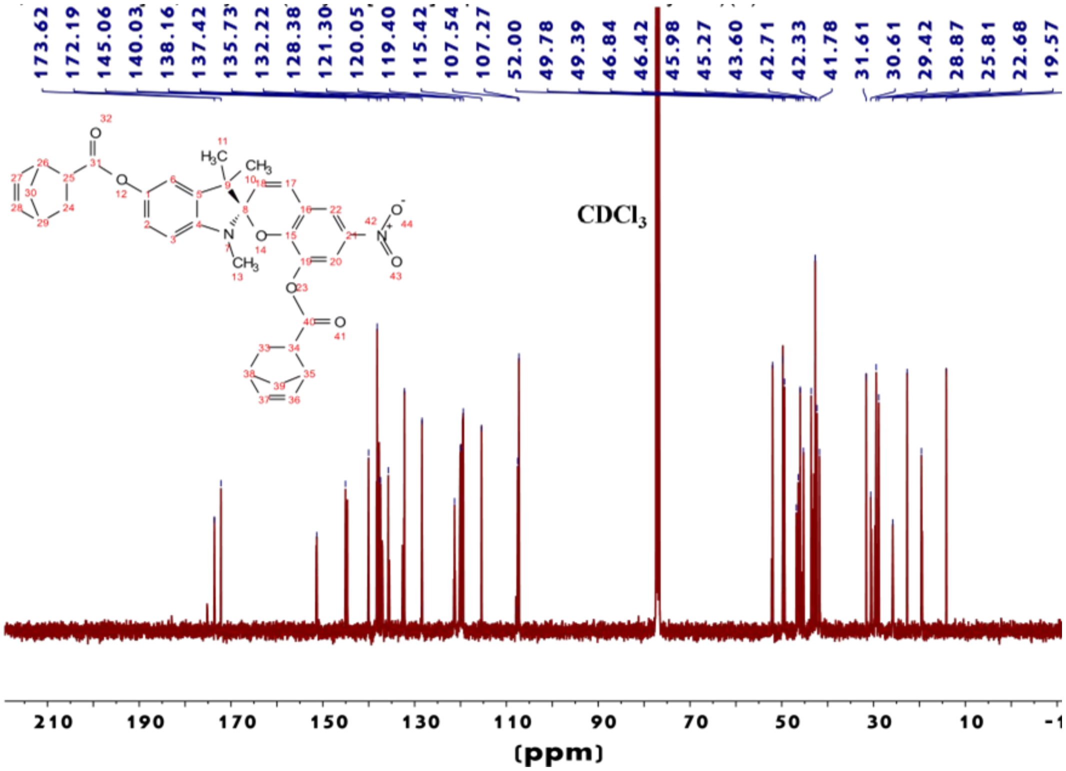

Figure S10: ^{13}C NMR spectra (101 MHz, $CDCl_3$-d_1) of (±)-1',3',3'-trimethyl-6-nitrospiro[chromene2,2'-indoline]-5',8-diyl bis(bicyclo[2.2.1]hept-5-ene-2-carboxylate)(**8**).

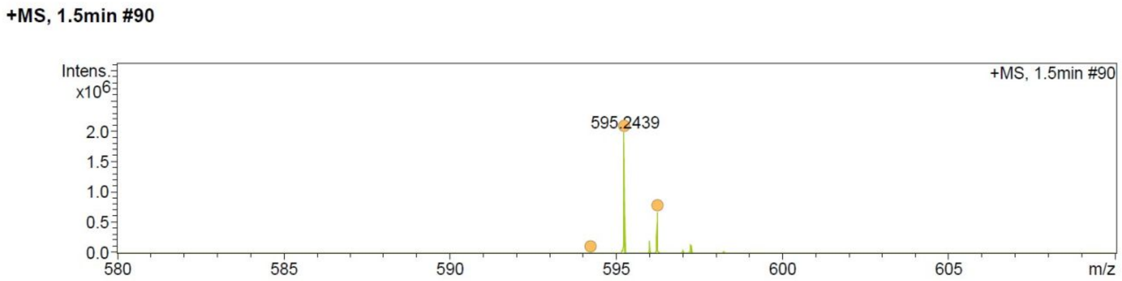

Figure S11: Mass spectra of (±)-1',3',3'-trimethyl-6-nitrospiro[chromene-2,2'-indoline]-5',8-diyl bis(bicyclo[2.2.1]hept-5-ene-2-carboxylate)(**8**) were obtained using an APCI source type.

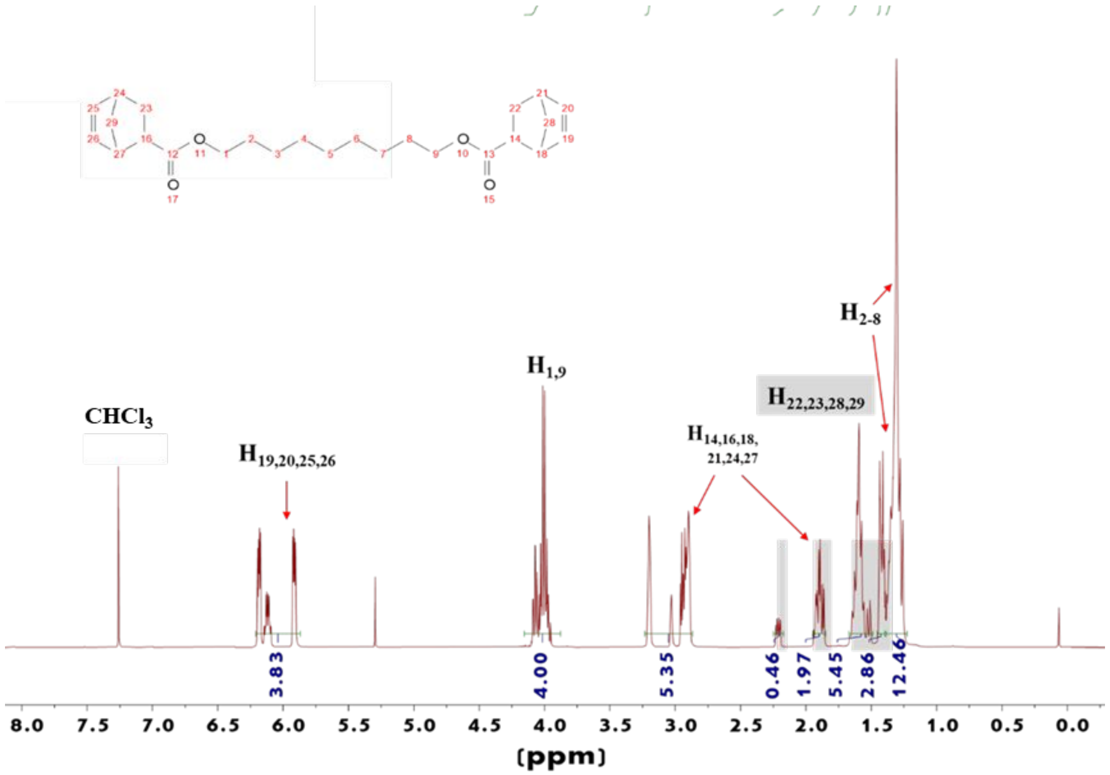

Figure S12: ^{1}H NMR spectra (400 MHz, $CDCl_3$) of nonane-1,9-diyl bis(bicyclo[2.2.1]hept-5-ene-2carboxylate)(**9**).

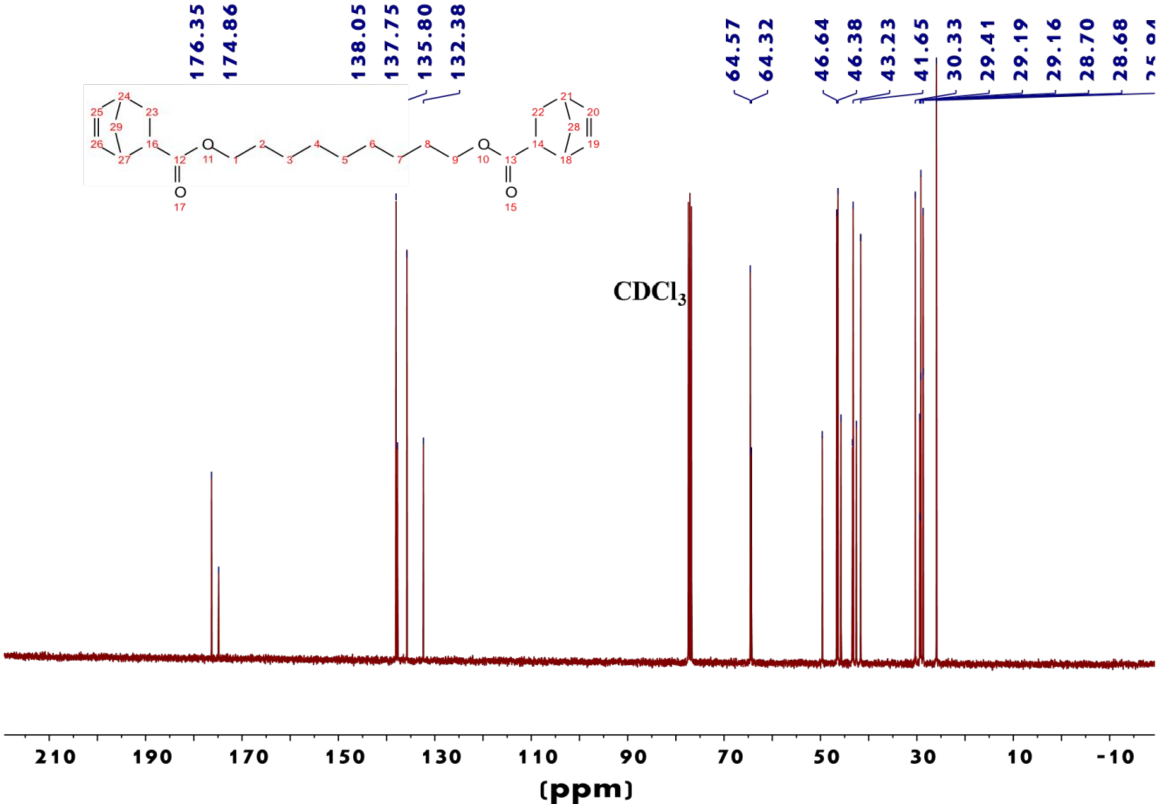

Figure S13: ^{13}C NMR spectra (101 MHz, $CDCl_3$) of nonane-1,9-diyl bis(bicyclo[2.2.1]hept-5-ene2-carboxylate)(**9**).

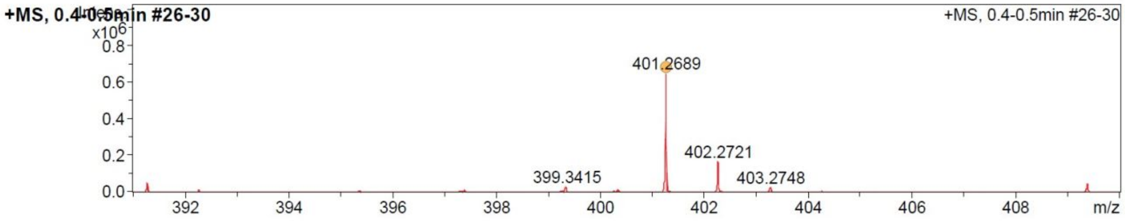

Figure S14: Mass spectra of nonane-1,9-diyl bis(bicyclo[2.2.1]hept-5-ene-2-carboxylate)(**9**) were obtained using an APCI source type.

3. Mechanophore application in Poly(styrene)

3.1 Synthesis and Characterizations

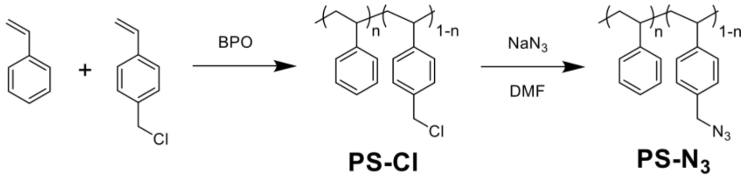

Scheme S6: Synthesis of **PS-Cl** and **PS-N$_3$**

Synthesis of PS-Cl

This copolymer was synthesized using conventional radical polymerization which has a commercial interest.[6] BPO (0.64 g, 2.48 mmol, 2.5% of monomer), VBC (2.8 mL, 19.8 mmol, 1 equiv), and styrene (9.1 mL, 79.42 mmol, 4 equiv) were added to a 25 mL two-necked Schlenk flask and sealed with a rubber septum. The polymerization was conducted under an argon atmosphere at 123 °C with magnetic stirring after 2 h the solution became viscous and the magnetic stirrer stopped moving. Then, the reaction mixture was diluted in 80 mL DCM and precipitated in 1 L MeOH thrice to give the **PS-Cl** (10 g, 88%) as a white powder.[7] ^{1}H NMR ($CDCl_3$) δ 6.14-7.21 (10H, Ph-H), 4.44.8 (2H, -CH_2Cl), 1.2-2.2 (3H, -$CHCH_2$-). M_n (THF-SEC): 30 KDa, Đ = 2.8. According to **Fig. S15a**, the molar percentage of **VBC** in the copolymer **PS-Cl** is 44%.[7]

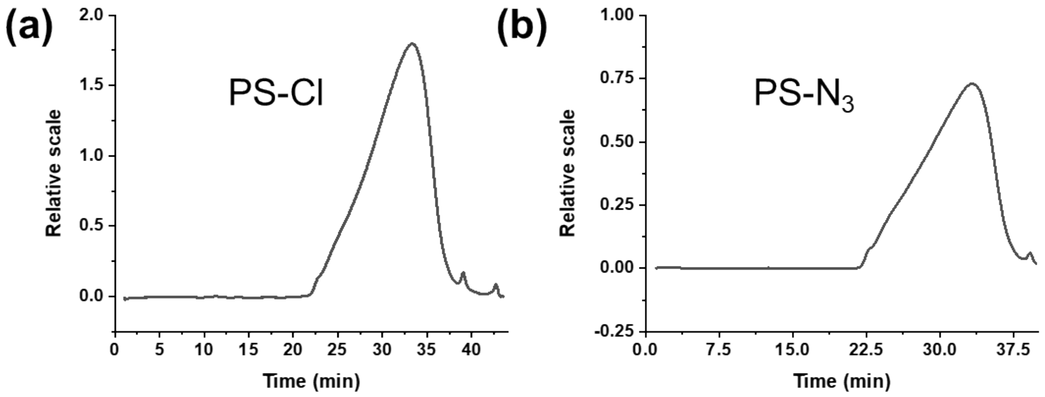

Figure S15: THF-GPC (signal from differential refractive-index detector) of linear precursor a| **PS-Cl**, M_w of 30 kDa, M_n of 10 kDa, Đ = 2.8. b| **PS-N$_3$** M_w of 36 kDa M_n of 9 kDa, Đ = 3.9. Molecular weights and molecular weight distributions were calculated by light scattering using a d_n/d_c value of 0.185 mL/g for poly(styrene).

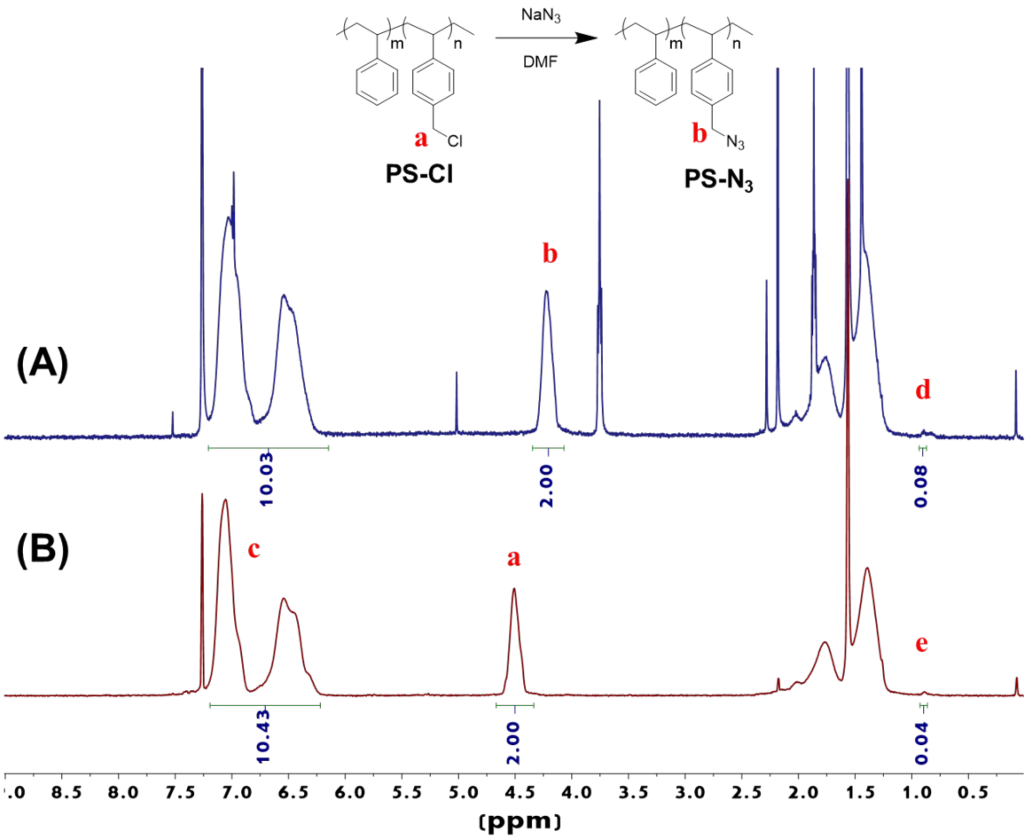

Figure S16: ^{1}H NMR (400 M, $CDCl_3$) spectra of a | **PS-Cl** and b | **PS-N$_3$**.

The molar ratio of the monomeric units F = [St]/[VBC] in random copolymers **PS-Cl** was calculated from ^{1}H NMR spectra according to the following equation:[7]

$$F = \frac{(I_c - 2 \times I_n)/5}{I_n/2}$$

where I_a is the integral value of the signals at 6.1-7.2 ppm attributed to the aromatic protons, and I_n is the integral value of the signal at 4.0-4.3 ppm attributed to the protons of chloromethylene group (–CH_2Cl) in the VBC unit. Which is

$$F = \frac{(10.43 - 2 \times 2)/5}{2/2} = 1.286$$

$$\begin{cases} \frac{[St]}{[VBC]} = F \\ [St] + [VBC] = 1 \end{cases}$$

$$\begin{cases} \frac{[St]}{[VBC]} = 1.286 \\ [St] + [VBC] = 1 \end{cases}$$

$$[St] = 1 - 0.44 = 0.56$$

$$[VBC] = 0.44$$

where [St] and [VBC] are molar parts of St 56 % and VBC 44 % units in a copolymer, respectively.

Synthesis of PS-N_3

PS-Cl (Mn = 30 kDa, 500 mg, 1 equiv) and sodium azide (1023 mg, 15.7 mmol, 20 equiv) were dissolved in DMF (10 mL) and stirred at room temperature for 48 h. The mixture was precipitated with ice-cooled water 2 times and dried in the air to given 335 mg (67% yield) as a white powder. M_n (THF-SEC): 36 KDa, Đ = 3.9.

Synthesis of spiropyran mechanophore linked poly(styrene) PS-Bis and PS-Mono and preparation of the dog-bone sample for tensile testing.

PS-Bis: **PS-N_3** (190 mg, 1.58 mmol, 1 equiv) and **Compound 8** (10 mg, 0.0168 mmol, 0.01 equiv) were dissolved in 10 mL of DCM, then the reaction was left at 50 °C for 3 days.

PS-Mono: **PS-N_3** (190 mg, 1.58 mmol, 1 equiv), **Compound 7** (8 mg, 0.0168 mmol, 0.01 equiv) and **Compound 9** (6.7 mg, 0.0168 mmol, 0.01 equiv) was dissolved in 10 mL of DCM, then the reaction was left at 50 °C for 3 days.

All the sample were left to dry in the dog-bone mold, and plasticized with 40 wt% DMF:1,4- dioxane (1:1 v/v) to remove the sample from the mold and perform the tensile measurements.

Calculation of mechanophore decoration in PS-Bis and PS-Mono

The mechanophore decoration in **PS-Bis** and **PS-Mono** is estimated based on the amount of norbornene consumed according to the following equation:

PS-Bis

Spiropyran functional amount is the same as crosslink density in **PS-Bis**.

Crosslink density in mol%:

$$\frac{Mol\ of\ crosslinks}{Mol\ of\ reactive\ site \times 100\%} = \frac{Mol\ of\ nonbornene\ groups \times 2}{Mol\ of\ reactive\ site \times 100\%}$$

$$= \frac{0.0168 \times 10^{-3} \times 2}{0.19 \div (0.44 \times 159 + 0.56 \times 104) \times 0.44 \times 100\%} = 5.136\%$$

Crosslink density in wt%:

$$\frac{g\ of\ crosslinks}{g\ of\ polymer \times 100\%} = \frac{10 \times 10^{-3}}{0.19 \times 100\%} = 0.52\%$$

PS-Mono

Spiropyran functional amount in wt% :

$$\frac{g\ of\ spiropyran}{g\ of\ polymer \times 100\%} = \frac{8 \times 10^{-3}}{0.19 \times 100\%} = 0.41\%$$

Crosslink density in mol%:

$$\frac{Mol\ of\ crosslinks}{Mol\ of\ reactive\ site \times 100\%} = \frac{Mol\ of\ nonbornene\ groups \times 2}{Mol\ of\ reactive\ site \times 100\%}$$

$$= \frac{0.0168 \times 10^{-3} \times 2}{0.19 \div (0.44 \times 159 + 0.56 \times 104) \times 0.44 \times 100\%} = 5.136\%$$

Crosslink density in wt%:

$$\frac{g\ of\ crosslinks}{g\ of\ polymer \times 100\%} = \frac{6.7 \times 10^{-3}}{0.19 \times 100\%} = 0.35\%$$

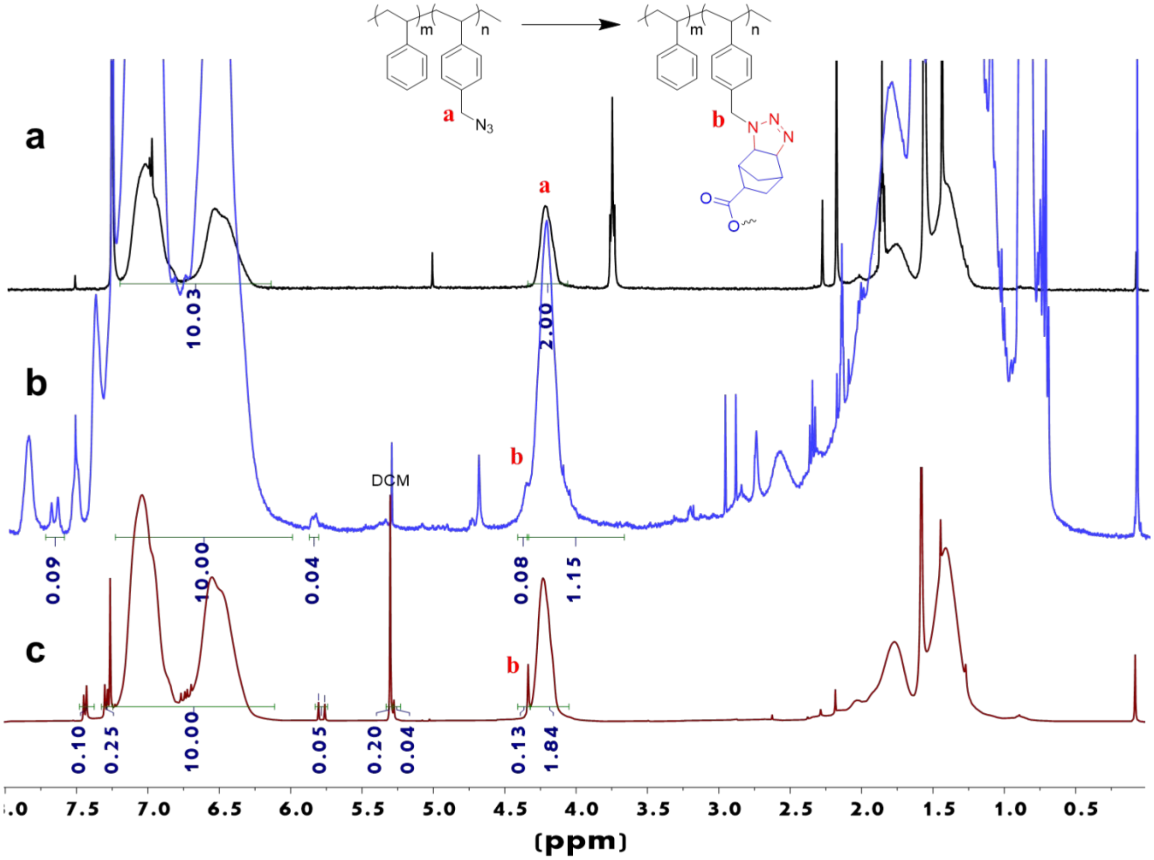

Figure S17: ^{1}H NMR spectra of **a | PS-N$_3$ b | PS-Mono** and **c | PS-Bis** in CDCl$_3$. **PS-Bis** is measured by the gel phase ^{1}H NMR.[8]

Synthesis of chain-centered SP-PS-N$_3$

The synthesis of chain-centered **SP-PS-N$_3$** was carried out following a modified experimental procedure.[1] SP-diIn was synthesized according to the established protocol.[2] In a 10 mL Schlenk tube, copper (I) chloride (5 mg), PMDETA (12 ul), and toluene (0.5 ml) were mixed under argon. After 5 min, SP-diIn [2] (25 mg) dissolved in toluene (1 mL) was added. Then, 4-azidomethylstyrene (0.15 mL) and styrene (0.9 mL) toluene were added to the reaction mixture. The reaction mixture was submitted to three freeze-pump-thaw cycles and then placed into an oil bath at 90 °C for 12 h. The reaction was

cooled producing a solid. The solid was slowly dissolved in $CHCl_3$ and passed through a pad of silica to remove the copper catalyst. The polymer solution was concentrated and precipitated into a large volume of diethyl ether. The slightly brown solid was dried under a vacuum. M_w (THF-MALS GPC): 6.5 kDa, PDI: 1.3.

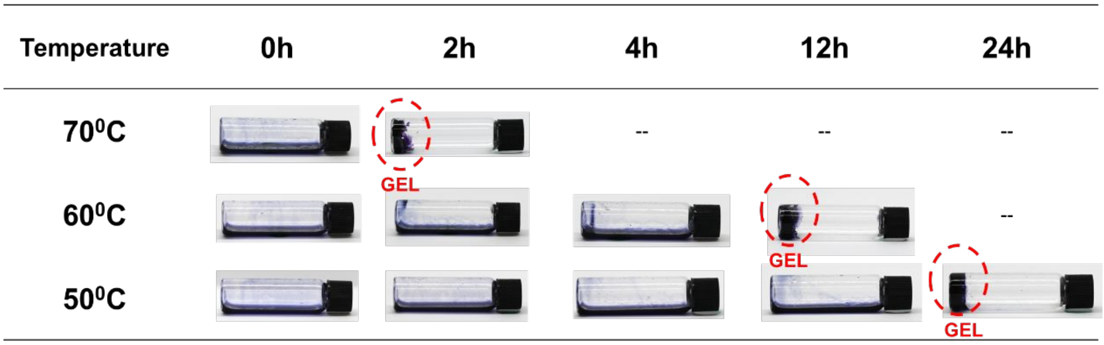

Figure S18: The formation of **PS-Bis** hydrogels, 91 mg **PS-N$_3$** with 15 mg **Compound 8** as crosslinker in 0.33 mL DCM (crosslinker amount 20 wt%) in a vertically 5 mL vial incubated under different temperatures. The picture was taken by placing the vial horizontally after incubation in the vertical position.

The heating process is necessary for the azide-norbornene Click Reaction to take place at reasonable time scales. Zhang et al. reported that the azide-norbornene polyaddition proceeded at room temperature and took 14 days to form gel networks, and over 3 months for the azide groups to completely consume.[9]

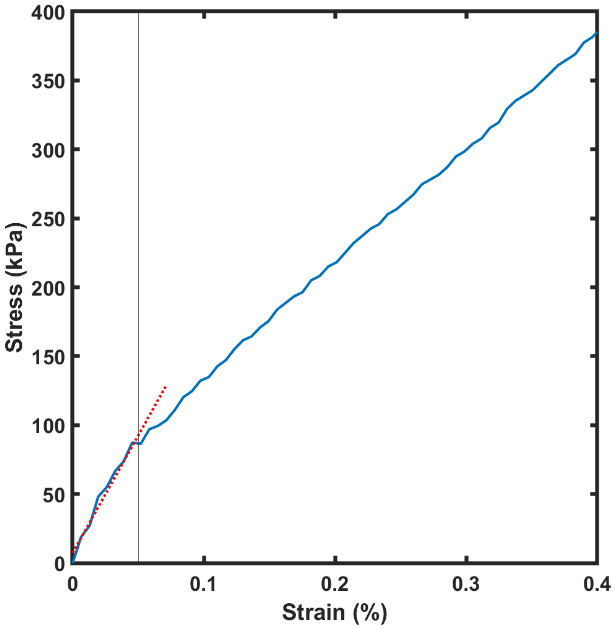

Figure S19: Stress-strain curve of the **PS-Bis** sample with a linear fit. 5% onset strain is on the edge of the linear regime.

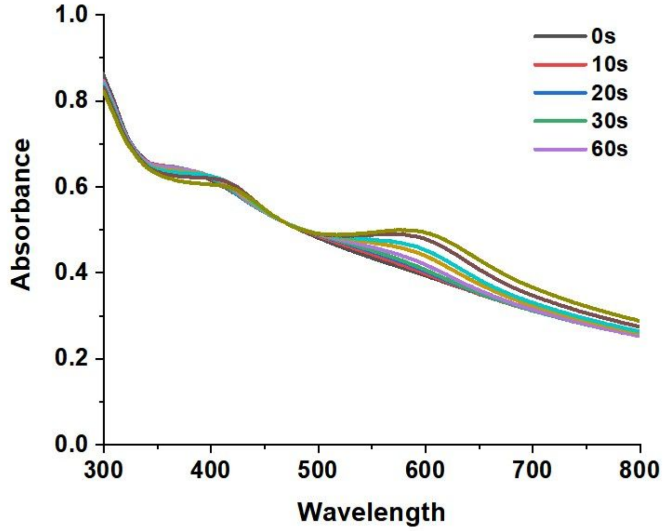

Figure S20: UV-Vis absorbance spectra of **PS-Mono** in MeCN solution (0.04 mg/mL) exposure in 16 W power ultraviolet light (254 nm wavelength) change over time.

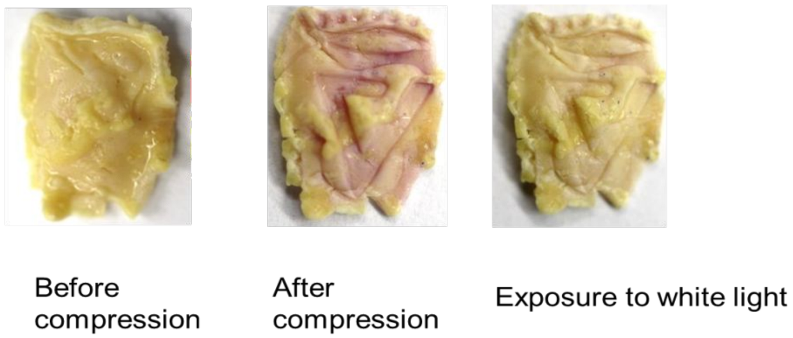

Figure S21: The images of **PS-Bis** before the press, after press, and after exposure in 5 min white light.

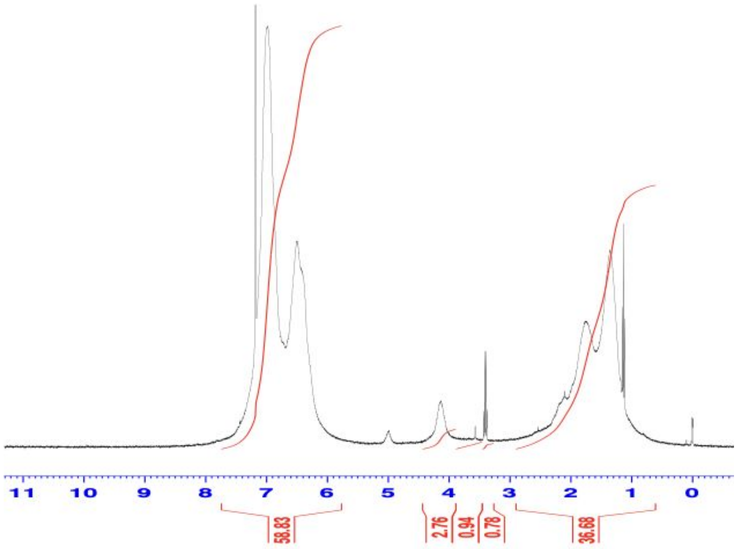

Figure S22: ^{1}H NMR spectra of chain-centered $\mathbf{SP\text{-}PS\text{-}N_3}$ in $CDCl_3$.

3.2 Application

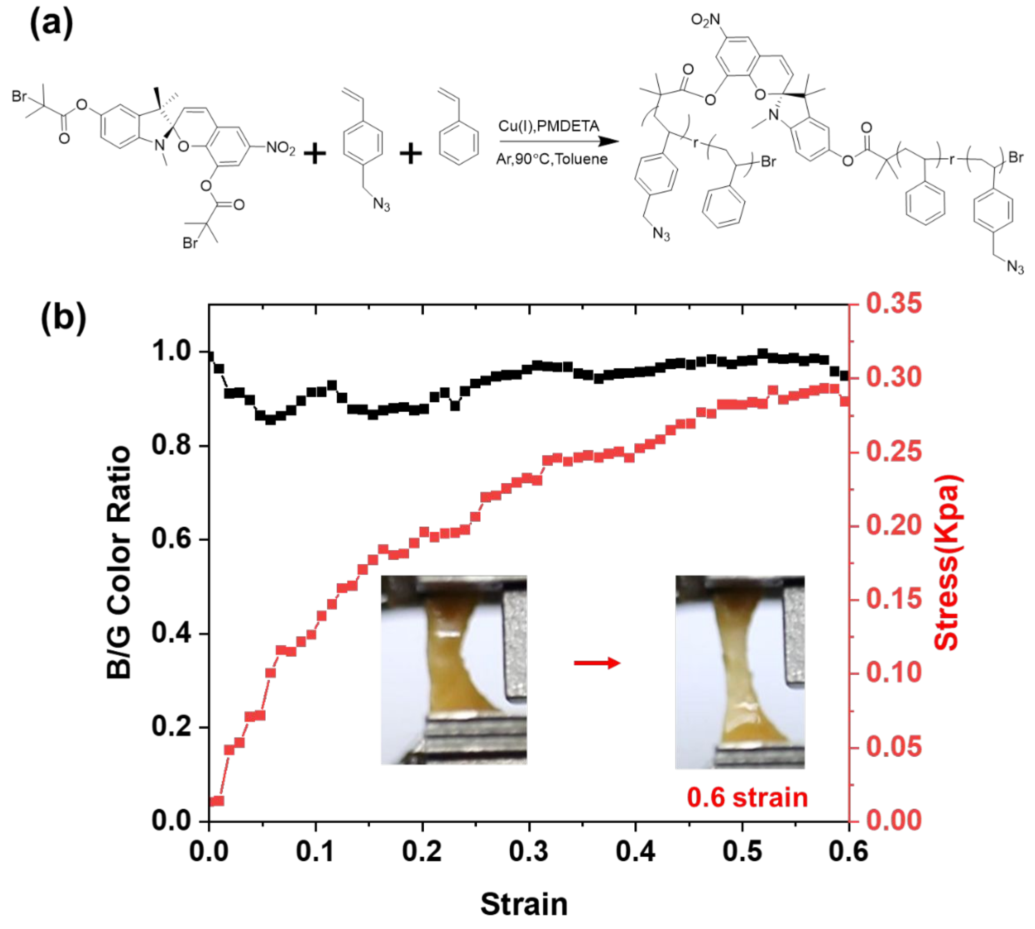

Figure S23: a| Synthesis of **SP-PS-N$_3$**. b|Uniaxial tensile testing via DMA coupled with video analysis shows mechanochemical activation of the polymerized spiropyran chain. The images are before tensile and after 60% strain. Stress-strain plot in dog-bone samples and colorimetric changes in the sample (Blue/Green ratio) measured along the intermediate level, normalized to 2 mm above the clamping area.

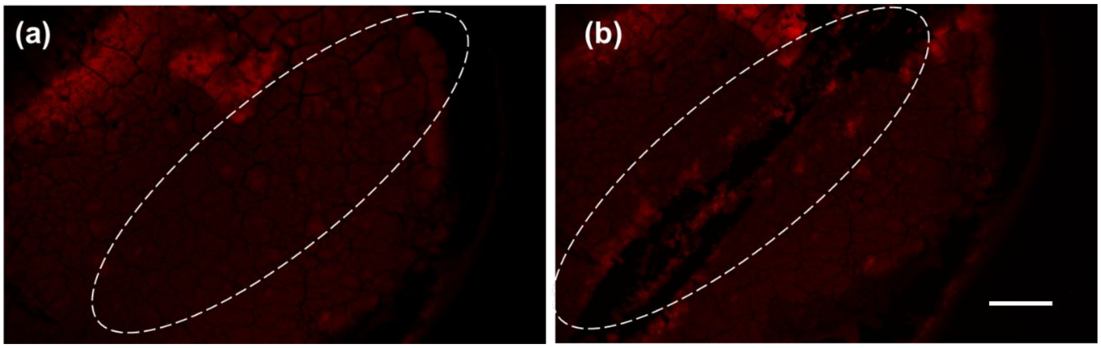

Figure S24: PS-SP DCM Droplets dried on glass slides a| and after cutting with a razor blade. b| The scale bar indicates 200 µm.

3.3 Comparison current method and our strategy

Table S1. A summary of monomer chemistries (previous method) and our strategy click reaction comparison.

Ref.	Catalyst	Solvent	Gas	Special treatment	Purification process
(10)	$CuBr_2$	DMSO	Argon	Three freeze pump-thaw cycles	Precipitated Cu(0) in 1:1 CH_3OH/H_2O
(2)	Cu(0) Me_6TREN	DMSO	Argon	Three freeze pump-thaw cycles	Filter, Precipitated in methanol
(11)	Dibutyltin dilaurate	THF	N_2	Three freezepump-thaw cycles	--
(12)	CuBr	THF	N_2	70 °C for 14 h	--
(13)		THF	N_2	70 °C for 2 days	Degassed, Removed solvent under a high vacuum
(14)	Sylgard 184 curing agent	xylene	-	70 °C for 4 h followed by 25 °C for 12 h	-
tetrazine-norbornene (This work)	--	Water	--	--	-
norbornene-azide (This work)	--	DCM	--	50°C 3d	-
norbornene-thiol (This work)	Benzoyl peroxide	Acetone	--	1h UV light	-

4. Mechanophore application in Alginate

4.1 Synthesis and Characterizations

Synthesis of ALG-Tetrazine.

The procedure follows previous work.[15] First, 133 mg sodium alginate was dissolved in 0.1 mol MES, 0.3 mol NaCl, pH 6.5 at 0.5% w/v buffer solution. Next, 0.6253 g EDC and 0.4635 g NHS were added to the alginate buffer solution, and then add 25 mg of Tetrazine into the solution. After stirring at room temperature for 24 h, the reaction was quenched by hydroxylamine and dialyzed for 4 days using dialysis tubing against a decreasing salt gradient from 0.15 mol to 0 mol NaCl in H_2O. The supernatant was freeze-dried after being centrifuged at 4000 rpm for 5 minutes to afford the **ALG-Tetrazine** (130 mg, 98%) as a pink solid. The alginate mechanophore mixture solution was shaken vigorously to mix for 2 min. During this process, it becomes turbid and viscous. The **ALG-Bis** hydrogel was left at room temperature for 8 h.

Determination of Tetrazine functional ratio in alginate.

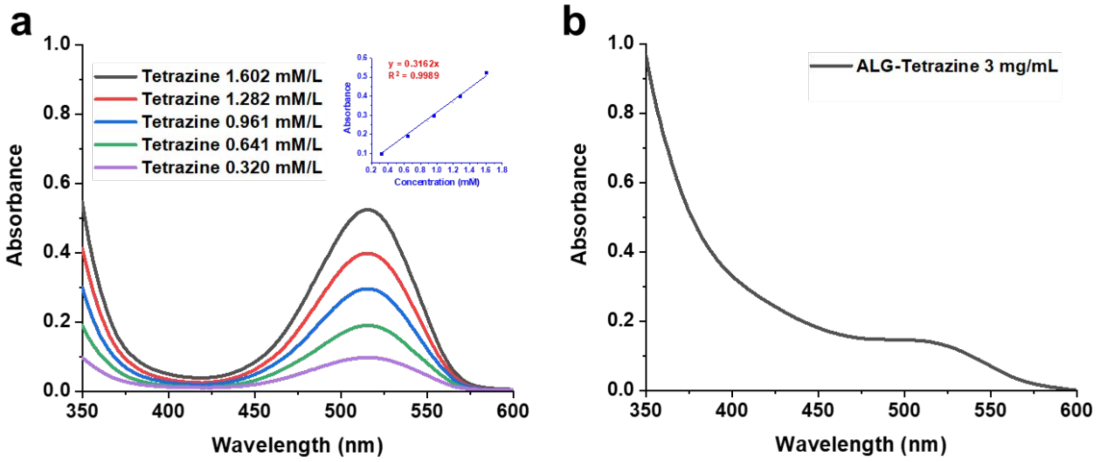

Figure S25: a | Tetrazine UV-vis standard curve and correlation coefficient in water, 3 mg/mL **ALG-Tetrazine** in water UV-vis absorption curve. **b |** The degree of tetrazine substitution of the available carboxylic acid groups of alginate is 2.82 % according to the absorbance at 520 nm wavelength.

Calculation of mechanophore decoration in Alginate

According to **Fig. S25** and **Fig. 27**, the degree of tetrazine substitution of the available carboxylic acid groups of alginate is 2.82 %. And 3 mg/mL ALG-Tetrazine containing $0.4255*10^{-3}$ mM/ml tetrazine. We assume that the crosslink density in mol is 100% according to ^{1}H NMR.

Crosslink density in wt%:

$$\frac{g\ of\ crosslinks}{g\ of\ polymer \times 100\%} = \frac{0.4255\times10^{-6}\div2\times594}{(0.003+Wsp)\ \times100\%} = \frac{0.126\times10^{-3}}{0.126\times10^{-3}+0.003}=4\ \text{wt\%}$$

Preparation of spiropyran mechanophore linked alginate ALG-Bis and ALG-Mono.

30 mg tetrazine alginate was fully dissolved into 2 mL H_2O, followed by the dropwise addition of 1 mL **Compound 8** or **Compound 7** acetone solution (8.4 mM/mL). The alginate solution was shaken vigorously during the mixing process. The **ALG-Bis** hydrogel were obtained at room temperature in less than 2 min.

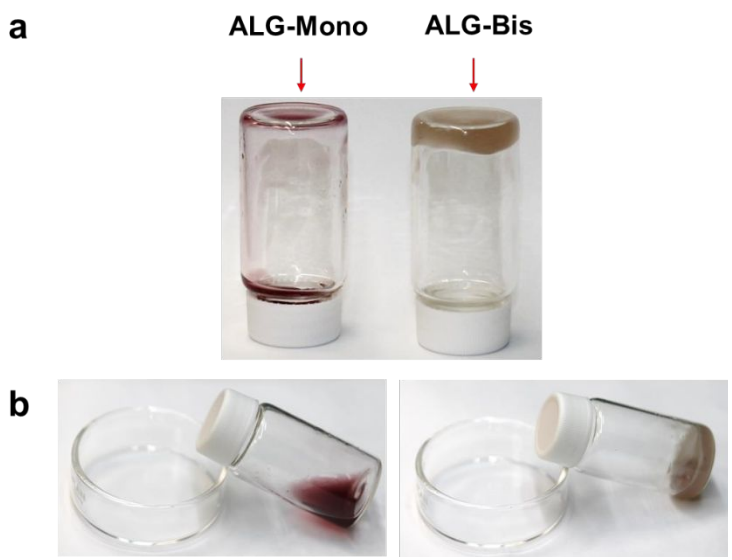

Figure S26: a | The images of 20 mg/mL **ALG-Mono** and **ALG-Bis** in aqueous solution upside down, and **b |** inclined at 45 °.

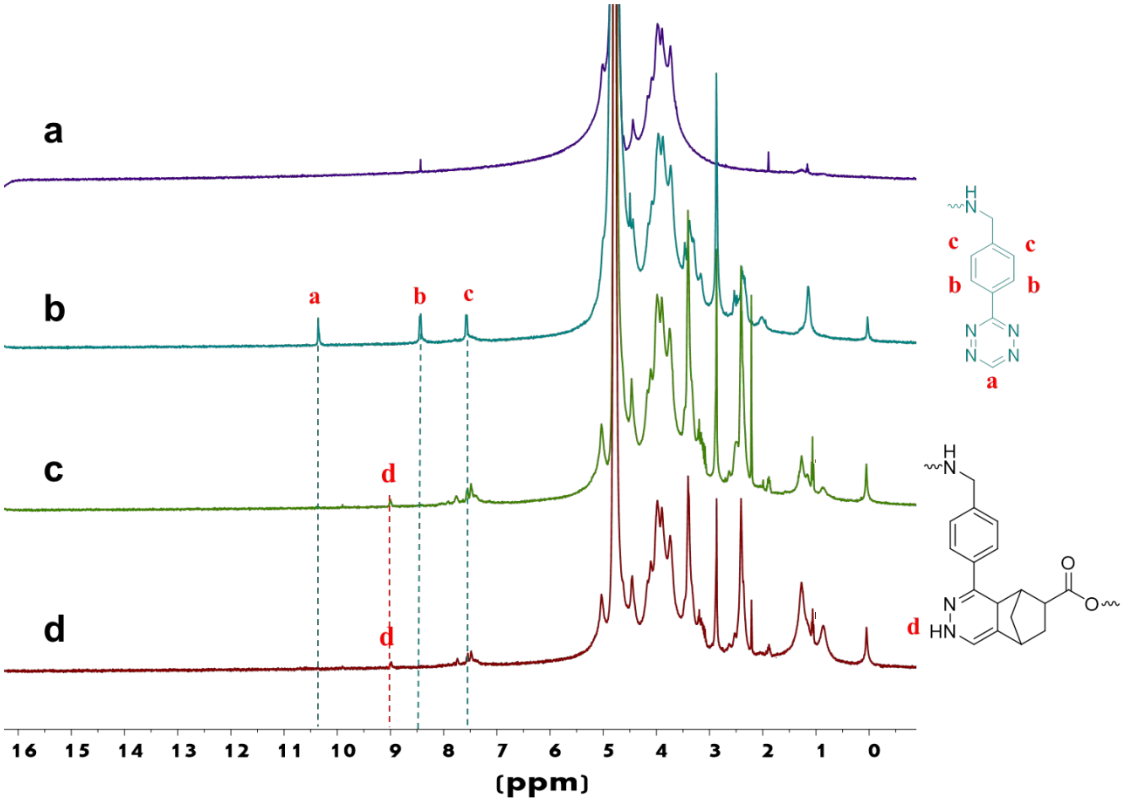

Figure S27: ^{1}H NMR (400 M, D_2O) spectra of 20 mg/mL a | alginate, b | **ALG-Tetrazine**, c | **ALG-Mono**, and d | **ALG-Bis**.

Synthesis of alginate-acrylamide film.

To enhance the mechanical properties of the spiropyran alginate film, the films were prepared as a composite film. According to previous work,[16] 31 mg **ALG-Tetrazine**, **ALG-Mono** or **ALG-Bis** was dissolved in 2 ml of a 12.4% acrylamide solution, respectively, followed by the addition of N, N-methylenebisacrylamide (0.0006 the weight of acrylamide) and ammonium persulphate (0.0004 of acrylamide). After vacuum degassing, add N, N, N′, N′-tetramethylethylenediamin (0.0025 the weight of acrylamide), mix with 1.5 % $CaSO_4$ (0.1328 of alginate), then inject into the mold. The hydrogel was cured with 16 W ultraviolet light (254 nm wavelength) at 50 °C for 45 min. After drying the film, the second polymerized network was formed by swelling the hydrogel with a 0.3 mL same concentration of acrylamide, N, N-methylenebisacrylamide, and ammonium persulphate solution, as first time curing.[17] Next, the hydrogel was subjected to a second time curing in the same condition under UV light. Afterward, the hydrogel was dried in a chemical hood for 1 day, transforming it into a film.

4.2 Application

Impact ALG-Bis spiropyran alginate film.

15 mg/mL **ALG-Bis** solution was injected into the round mold (internal diameter: 1.9 cm, height: 1 cm) and dried to a film. Then manually impact it using a scissor. After that, expose the film under white light for 1 hour. Fluorescence analysis before and after impact was studied using a fluorescence microscope (Nikon ECLIPSE Ts2R) to follow the mechanochromic behavior and recovery behavior on alginate film.

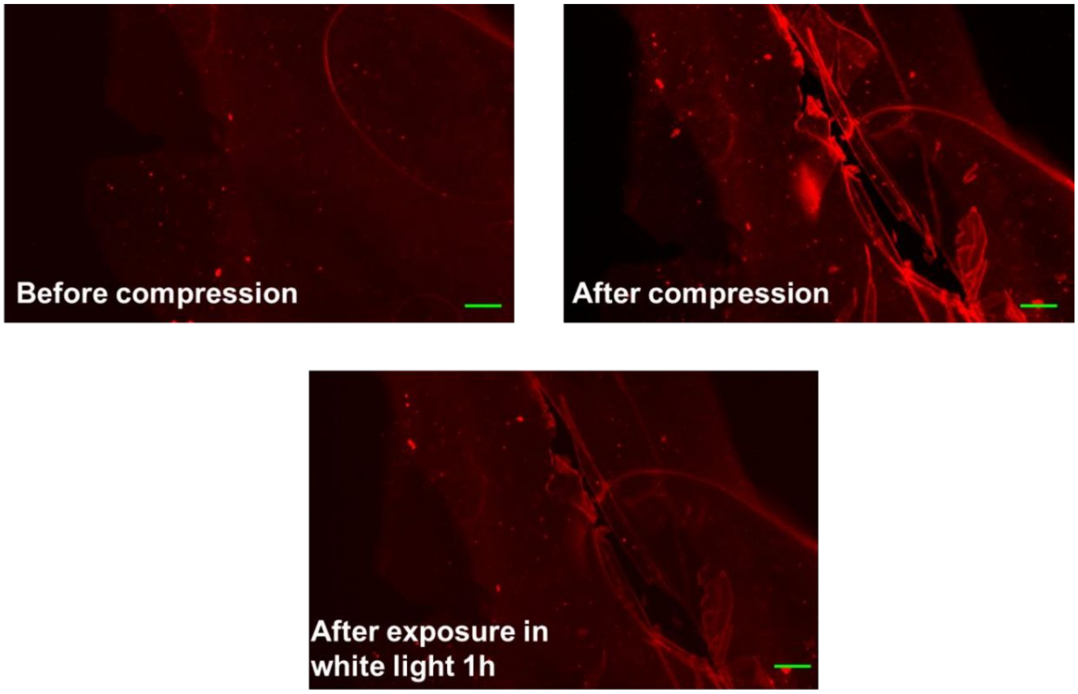

Figure S28: ALG-Bis film before the compression, after compression, and after exposure to white light for 1h under a red fluorescent protein (RFP) filter using a fluorescence microscope. The scale bar indicates 200 μm.

Folding alginate-acrylamide film.

Before the folding experiment, the **ALG-Bis** alginate-acrylamide film was rehydrated to 40 wt% water as a hydrogel thin film. The **ALG-Bis** alginate-acrylamide hydrogel is folded between glass slides at varying angles. Use two pieces of glass to sandwich a portion of the film and secure it, then use another two pieces of glass to sandwich and fix the remaining portion of the film. Fold the film inward along the gap between the glass pieces at various angles.

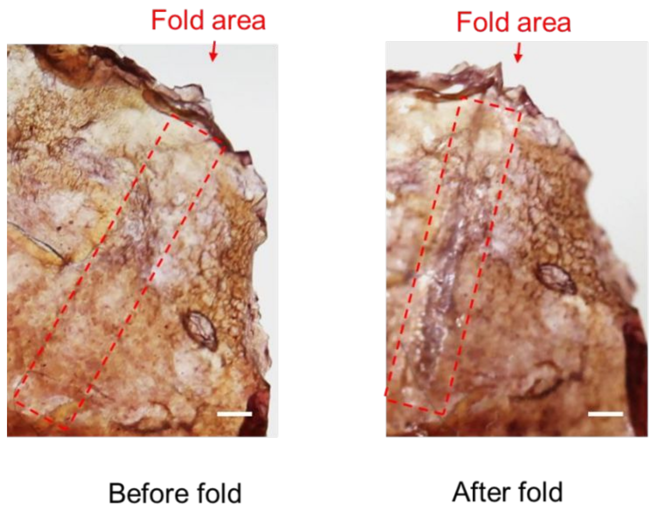

Figure S29: Digital photograph of **ALG-Bis** alginate-acrylamide film before and after bending 180°. The scale bar indicates 300 μm.

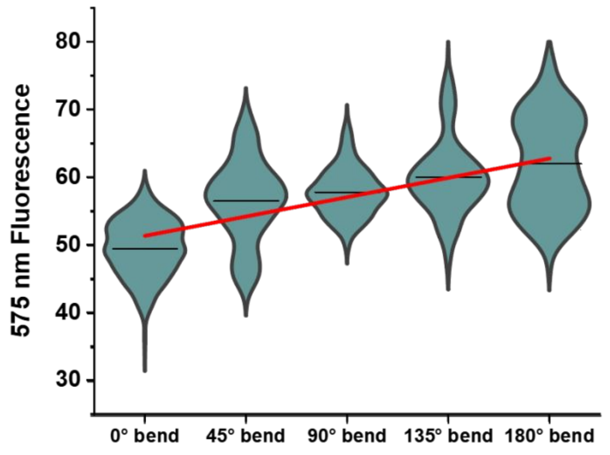

Figure S30: Fluorescence intensity at 575 nm of **ALG-Bis** alginate-acrylamide film under various bending angles. The obtained data were plotted, with the eye guideline represented by a red line.

4.3 FEA modeling

4.3.1 Context

The finite element model was designed to determine the stresses a rectangular film of **ALG-Bis** alginate-acrylamide film undergoes while incrementally folded from a flat configuration to a fully folded one. This stress is then correlated with the activation or not of the spiropyran molecules and thus, determines the folding energy needed to activate them.

4.3.2 Geometry

Experimentally, we attached a 10x5x0.5 mm^3 alginate film to two glass slides and folded them incrementally, one on the other, at different angle values measured by a protractor. The slides leave a gap of 3 mm to allow free movement of the film during the folding (**Fig. S31a**). To reduce computation time, we simplified the problem to a 5x5x0.5 mm^3 square with a symmetry plane, as shown in **Fig. S31b**, as the geometry is symmetrical to the folding plane.[18]

4.3.3 Boundary, contact and loading conditions

During the quasi-static simulations, lateral and top surfaces were free of displacement constrain, and boundary conditions were applied on one edge of the model corresponding to the folding plane and the bottom face corresponding to the displacement of the glass slide. The symmetry plane is composed of one fixed node at the edge, a sliding line at the plane's base, and symmetric nodes on the rest of the plane.

A displacement loading was applied to the nodes corresponding to the interface between the glass slide and the film i.e. all the nodes were distant of 1.5 mm from the symmetry plane. After translating the center of rotation at $y = Y + e$ where e is the thickness of the film and Y the initial y coordinates and y the y coordinates after translation, we applied the following displacement load:

$$\begin{cases} yn = -x_n & \text{for } \theta = 90 \\ y_n = x_n(\cos\theta - 1) & \text{for } \theta < 90 \end{cases}$$

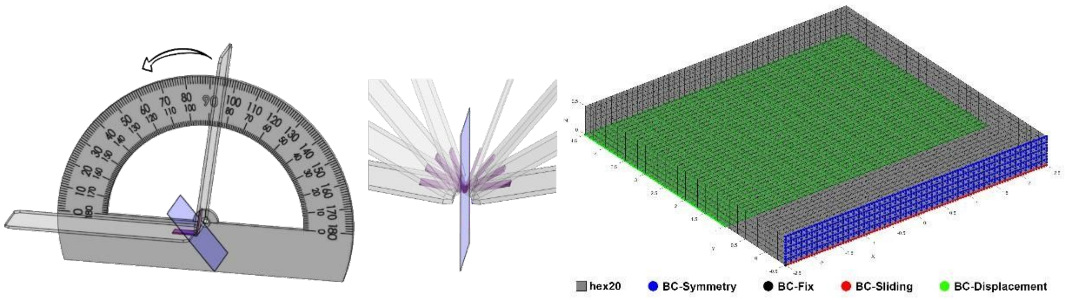

Figure S31: a | experimental setup of folding the film: the film is attached to two glass slides and folded on itself by moving the glass slides closer one to the other. The folding angle is measured by a protractor. b | The planar symmetric FE model was used in the force study. The half-film is represented by a square with a symmetry plan. 3D view of the uniform mesh made of quadratic hexahedral (hex20) elements.

4.4.4 Computational simulations and outcome measures

To determine the Von Mises stress and standard energy density (SED) required to activate mechanophores, we developed a finite element model of the folding using the opensource FE Pardiso solver FEBio version 4.2[18] via the GIBBON open-source toolbox version 3.5.0[19] used with MATLAB 9.14 R2023a (The Mathworks Inc., Natick, MA, USA) for pre and post-processing of the simulations The runtime of each simulation was in the range of 2 minutes for small folding to 20 min for the fully folded model using a 64-bit Windows 10 pc with an Intel(R) Xeon(R) W-2245 CPU @ 3.90 GHz and 128 GB RAM.

Mechanical properties of the ALG-Bis alginate-acrylamide film used have been determined by using a 3345 Universal testing machine (Instron, Norwood MA, USA) mounted with a 2530-5N load (Instron, Norwood MA, USA) and 2711 micro grips (Instron, Norwood MA, USA). 12*10*0.5 mm^3 samples were extended at a rate of 10 mm/min. Young's modulus of the film is determined by fitting the strain-stress curve up to 30% strain with a linear model using Matlab's fit function. We found E= 248 kPa **(Fig. S32**). The Poisson's ratio is fixed at 0.49 as the films are assumed to be incompressible.

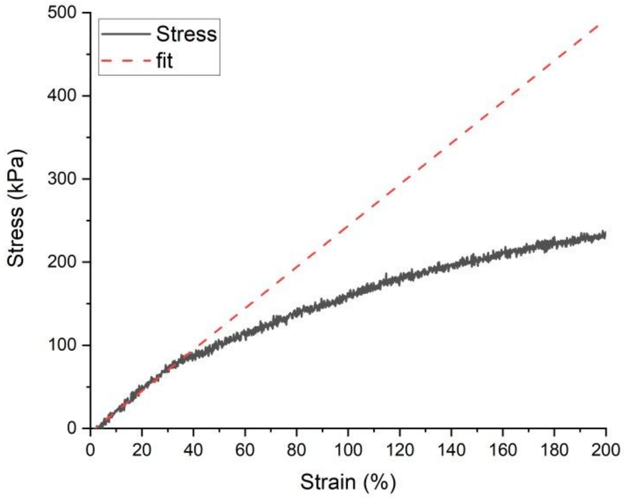

Figure S32: Strain-stress traction curve of **ALG-Bis** alginate-acrylamide film.

The meshing of the model into quadratic hexahedral (hex20) elements was performed using the meshing tool provided by FEBio. A convergence study has been done before running all the simulations to find the minimal ammount of elements and, thus, the shortest simulation time, leading to a constant solution. The parameter used in the simulation is the distance between the nodes. Only a few values of distances between nodes allowed to have an entire number of elements both in x, y and z directions, as presented in **Table S2**.

Table S2. Number of elements used for calculating the stress during the folding of the alginate-spyropiran film.

Distance between nodes	Number of elements over x, y	Number of elements over z	Total number of elements
0.5	10	1	100
0.25	20	2	800
0.125	40	4	6400
0.1	50	5	12500
0.0625	80	8	51200
0.05	100	10	100000

Fig. S33 shows the values of Effective strain (Von Mises stress) and calculation time over the number of elements for a folding angle of 100°. We see that from 6 400 elements, the solution differs only by 2.5 % from the best value we got and that for 12 500 elements, the solution differs only by 1.6%. However, the simulation time exponentially increases with the number of elements increasing from a few hundred seconds (346 s) at 12 500 elements to close to one hour (3347 s) for 51 200 elements without a significant increase in the accuracy of the simulation. This is why we ran our simulations with 12 500 elements.

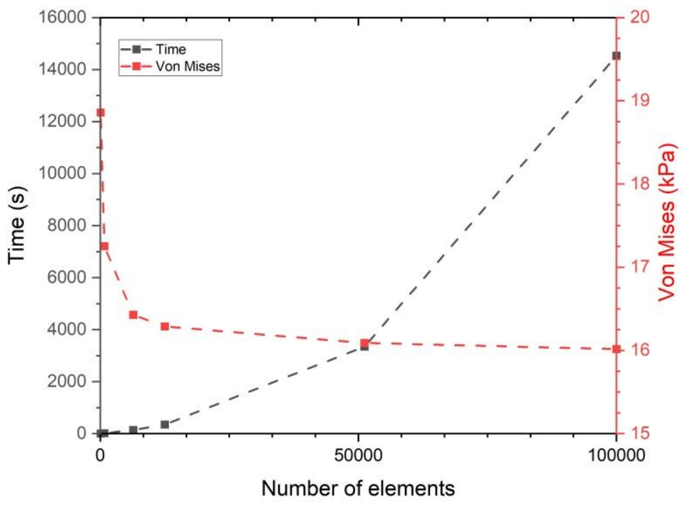

Figure S33: Values of effective strain (von Mises stress) and calculation time over the number of elements for a folding angle of 100°.

The stress is the average VonMises stress on all the thicknesses of the gel obtained by the following equation:[20]

$$vonMiseskPa = \frac{1}{n}\sum\sqrt{\frac{1}{2}\left((s_1-s_2)^2 + (s_2-s_3)^2 + (s_3-s_1)^2\right)}\cdot 1000$$

\begin{equation}

vonMiseskPa = \frac{1}{n}\sum\sqrt{\frac{1}{2}\left((s_1-s_2)^2 + (s_2-s_3)^2 +(s_3-s_1)^2 \right)}\cdot 1000

\end{equation}

Where s_i are the principal stresses.

We used the term "bending strain" to refer to the maximum folding strain at the central plane of the sheet. (strainStressData(n/5+1,2) = max(max(N_strain_mat(:,:,10))*100); (c.f. matlab files of simulation.)

Results

To determine the energy needed to activate by folding spiropyran molecules, we plotted the volumetric distribution of the effective (von Mises) stresses and SED over the folding angle of the film (**Fig. S34**).

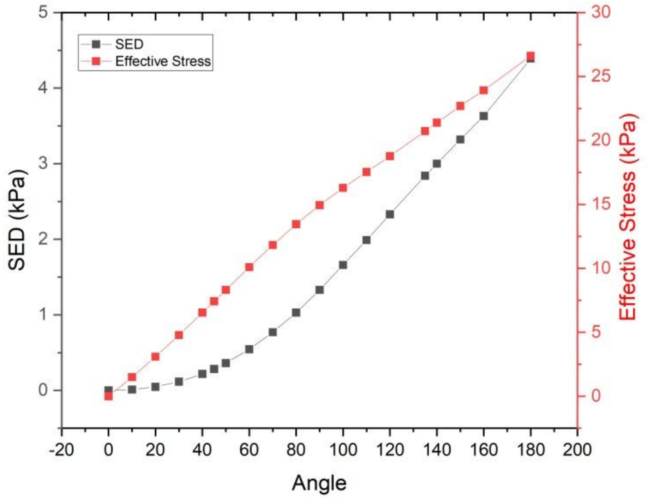

Figure S34: Volumetric distribution of the effective (von Mises) stresses and SED over the folding ang-le of the film.

Both SED and effective stress increase while the film is folded on itself (**Fig. S35**).

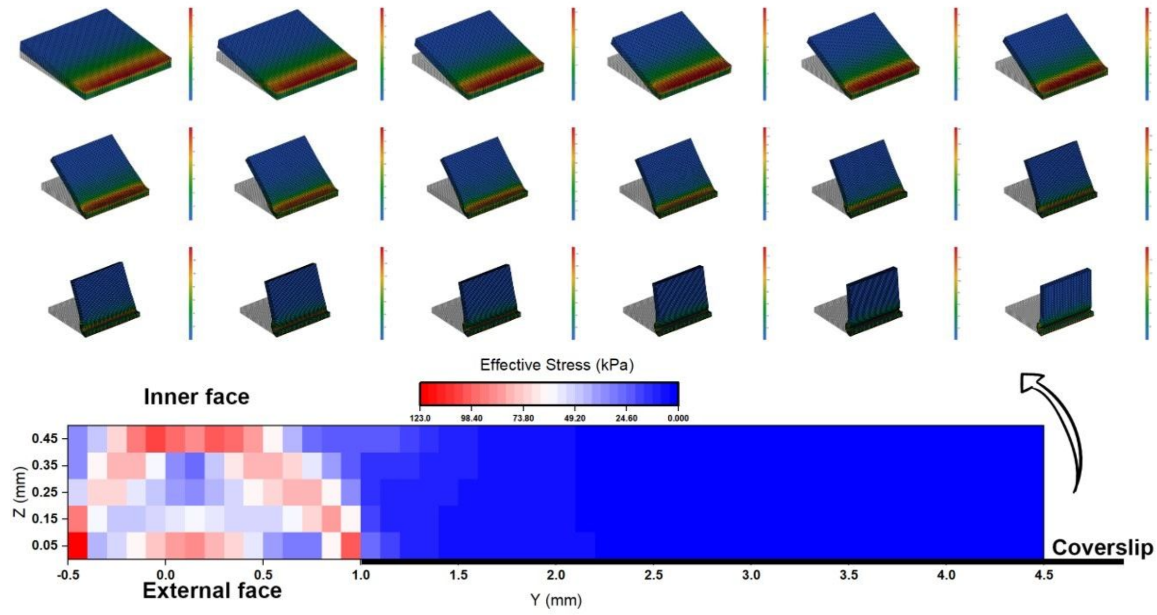

Figure S35: Effective stress in the film.

Fig. S35 top shows the different steps of the film folding, where we see the apparition of a bulge at the basis of the film, in the very high folding angle values, suggesting the heterogeneous distribution of the effective stress in the film. This is confirmed by the slice view on the Y-Z plane (x=0, center of the film), where we notice that the maximal stresses are encountered at the base of the folding plane, at the contact point between the coverslip and the film, and finally at the most compressed and extend areas of the bulge.

5. Mechanophore application in Wool

5.1 Synthesis and Characterizations

Preparation of Wool-Bis

The wool fibers were pretreated by MPTES according to an established protocol.[21] Wool fibers were purified by extraction with acetone in a soxhlet extractor for 24 h followed by washing with cold water and air drying, to remove the dirt and surface lipids before use. Then, 200 mg wool fibers were treated with 5 mL detergent solution containing 15g/L NaOH at 70 °C for 20 min to further remove grease and acquire some thiol group, which provided a favorable basis for the subsequent process. After washing the fiber with water and air drying, the fiber was immersed into 1 mL as washed 5% wt MPTES at ethanol-water v/v = 4:1 solution for 3 min and cured in a vacuum at 110 °C for 4.5 min followed by washing with cold water and air drying. Then **Compound 8** was dissolved in acetone at a ratio of 1:50, and 0.5 % wt BPO was added as a photo-initiator. Subsequently, the MPTES-modified wool fiber was dipped into the mixture solution and irradiated under UV light for 1 h. Upon completion of the reaction, the wool fiber was left immersed in solution for 12 h. Then the surface of the fiber was cleaned with water and dried in the air to obtain the final product.

Determination thiol group in wool

The amount of thiol group in the wool during the functional process was determined by measuring the absorbance of wool soak solution in Ellman's reagent at 412 nm wavelength. Prepare 0.8 mM, 0.4 mM, 0.2 mM, and 0.1 mM standard MPTES solution or wool fiber in the buffer solution containing 0.1 M sodium phosphate, 1 mM EDTA in pH 8.0. Prepare 4 mg/mL 5,5-dithio-bis-(2nitrobenzoic acid) in buffer solution as Ellman solution. Mix 0.25 mL standard MPTES solution or sample immersed solution, 2.5 mL buffer solution, and 50 uL Ellman solution, and incubate at room temperature for 15 min. Use the UV-vis spectra to take a full absorbance spectrum of the solution.

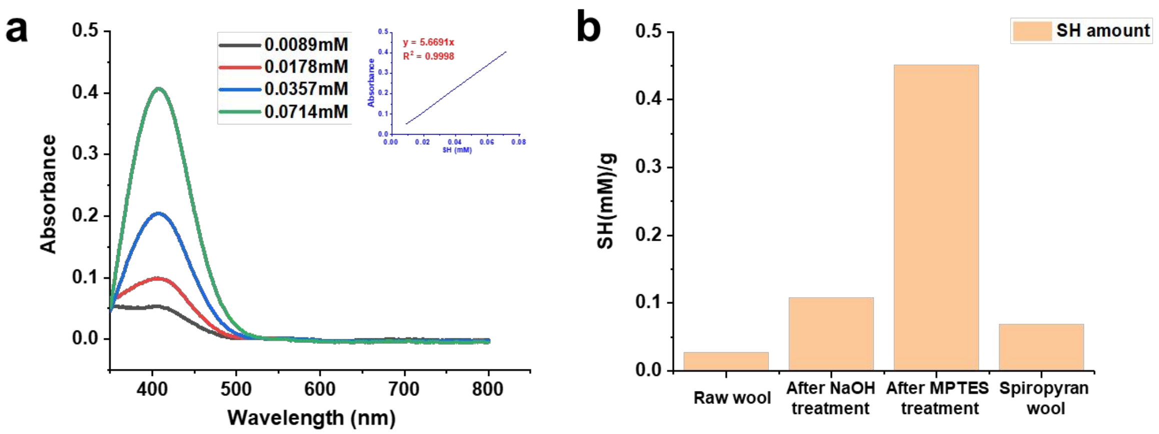

Figure S36: a | The UV-vis spectra of MPTES at various concentrations by reacting with Ellman solution and their correlation coefficient. **b |** The thiol content in the wool in different functional processes.

Evaluation of spiropyran functional ratio on wool

The evaluation of the spiropyran functional ratio on wool using FTIR (Fourier Transform Infrared Spectroscopy) is based on establishing a linear relationship between absorbance and the amount of bis-norbornene spiropyran in the 200 mg of KBr pellet at the 2960 cm^{-1} wavenumber. By measuring the absorbance of the bis-norbornene spiropyran wool sample, its spiropyran content can be determined by referring to the established linear relationship. According to **Fig. S32**, the mass percentage of spiropyran in the spiropyran wool is 6.7 wt%.

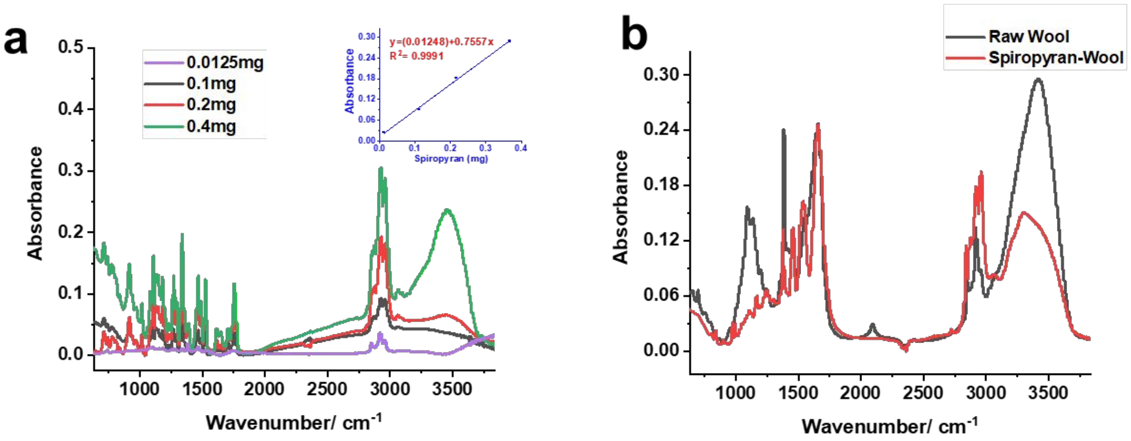

Figure S37: a | IR spectroscopy of various amounts of **Compound 8** pelleted with 200 mg KBr and their correlation coefficient at 2960 cm^{-1} wavenumber. **b** | IR spectra of raw wool and spiropyran functionalized wool.

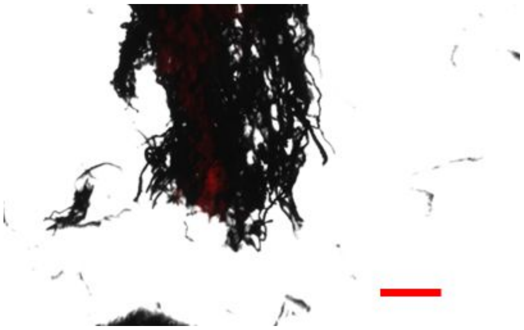

Figure S38: **SP wool** activated by scissor cutting. The scale bar indicates 200 μm.

6. Supplemental References

(1) Trejo-Maldonado, M.; Elizalde, L. E.; Le Droumaguet, B.; Grande, D. Synthesis of Triazole-Functionalized Diblock Copolymers as Templates for Porous Materials. *React. Funct. Polym.* **2021**, *164*, 104919.

(2) Davis, D. A.; Hamilton, A.; Yang, J.; Cremar, L. D.; Van Gough, D.; Potisek, S. L.; Ong, M. T.; Braun, P. V.; Martínez, T. J.; White, S. R.; Moore, J. S.; Sottos, N. R. Force-Induced Activation of Covalent Bonds in Mechanoresponsive Polymeric Materials. *Nature* **2009**, *459* (7243), 68–72.

(3) Potisek, S. L.; Davis, D. A.; Sottos, N. R.; White, S. R.; Moore, J. S. Mechanophore-Linked Addition Polymers. *J. Am. Chem. Soc.* **2007**, *129* (45), 13808–13809.

(4) Kim, T. A.; Robb, M. J.; Moore, J. S.; White, S. R.; Sottos, N. R. Mechanical Reactivity of Two Different Spiropyran Mechanophores in Polydimethylsiloxane. *Macromol.* **2018**, *51* (22), 9177–9183.

(5) Simpson, N.; Takwa, M.; Hult, K.; Johansson, M.; Martinelle, M.; Malmström, E. Thiol-Functionalized Poly(ω-Pentadecalactone) Telechelics for Semicrystalline Polymer Networks. *Macromol.* **2008**, *41* (10), 3613–3619.

(6) Puts, G.; Venner, V.; Améduri, B.; Crouse, P. Conventional and RAFT Copolymerization of Tetrafluoroethylene with Isobutyl Vinyl Ether. *Macromol.* **2018**, *51* (17), 6724–6739.

(7) Willdorf-Cohen, S.; Zhegur-Khais, A.; Ponce-González, J.; Bsoul-Haj, S.; Varcoe, J. R.; Diesendruck, C. E.; Dekel, D. R. Alkaline Stability of Anion-Exchange Membranes. *ACS Appl. Energy Mater.* **2023**, *6* (2), 1085–1092.

(8) Nijem, S.; Song, Y.; Schwarz, R.; Diesendruck, C. E. Flex-Activated CO Mechanochemical Production for Mechanical Damage Detection. *Polym. Chem.* **2022**, *13* (27), 3986–3990.

(9) Zhang, X.; Zhang, Q.; Wu, Y.; Feng, C.; Xie, C.; Fan, X.; Li, P. Polyaddition of Azide‐Containing Norbornene‐Based Monomer through Strain‐Promoted 1,3‐Dipolar Cycloaddition Reaction. *Macromol. Rapid Commun.* **2016**, *37* (16), 1311–1317.

(10) Wang, L.-J.; Zhou, X.-J.; Zhang, X.-H.; Du, B.-Y. Enhanced Mechanophore Activation within Micelles. *Macromol.* **2016**, *49* (1), 98–104.

(11) Zhang, H.; Chen, Y.; Lin, Y.; Fang, X.; Xu, Y.; Ruan, Y.; Weng, W. Spiropyran as a Mechanochromic Probe in Dual Cross-Linked Elastomers. *Macromol.* **2014**, *47* (19), 6783–6790.

(12) Jiang, S.; Zhang, L.; Xie, T.; Lin, Y.; Zhang, H.; Xu, Y.; Weng, W.; Dai, L. Mechanoresponsive PS-PnBA-PS Triblock Copolymers via Covalently Embedding Mechanophore. *ACS Macro Lett.* **2013**, *2* (8), 705–709.

(13) Lee, C. K.; Beiermann, B. A.; Silberstein, M. N.; Wang, J.; Moore, J. S.; Sottos, N. R.; Braun, P. V. Exploiting Force Sensitive Spiropyrans as Molecular Level Probes. *Macromol.* **2013**, *46* (10), 3746–3752.

(14) Kim, D. W.; Medvedev, G. A.; Caruthers, J. M.; Jo, J. Y.; Won, Y.-Y.; Kim, J. Enhancement of Mechano-Sensitivity for Spiropyran-Linked Poly(Dimethylsiloxane) via Solvent Swelling. *Macromol.* **2020**, *53* (18), 7954–7961.

(15) Desai, R. M.; Koshy, S. T.; Hilderbrand, S. A.; Mooney, D. J.; Joshi, N. S. Versatile Click Alginate Hydrogels Crosslinked via Tetrazine–Norbornene Chemistry. *Biomaterials* **2015**, *50*, 30–37.

(16) Sun, J.-Y.; Zhao, X.; Illeperuma, W. R. K.; Chaudhuri, O.; Oh, K. H.; Mooney, D. J.; Vlassak, J. J.; Suo, Z. Highly Stretchable and Tough Hydrogels. *Nature* **2012**, *489* (7414), 133–136.

(17) Chen, Y.; Yeh, C. J.; Qi, Y.; Long, R.; Creton, C. From Force-Responsive Molecules to Quantifying and Mapping Stresses in Soft Materials. *Sci. Adv.* **2020**, *6* (20), eaaz5093.

(18) Moerman, M.; Gibbon, K. The Geometry and Image-Based Bioengineering Add-On. *JOSS* **2018**, *3* (22), 506.

(19) Maas, S. A.; Ellis, B. J.; Ateshian, G. A.; Weiss, J. A. FEBio: Finite Elements for Biomechanics. *J. Biomech. Eng.* **2012**, *134* (1), 011005.

(20) Salençon, J. *Handbook of Continuum Mechanics*; Springer Berlin Heidelberg: Berlin, Heidelberg, **2001**.

(21) Fan, J.; Bao, B.; Wang, Z.; Xu, R.; Wang, W.; Yu, D. High Tri-Stimulus Response Photochromic Cotton Fabrics Based on Spiropyran Dye by Thiol-Ene Click Chemistry. *Cellulose* **2020**, *27* (1), 493–510.
